# Human Cytomegalovirus miR-US33as-5p Targets IFNAR1 to Achieve Immune Evasion During Both Lytic and Latent Infection

**DOI:** 10.3389/fimmu.2021.628364

**Published:** 2021-03-05

**Authors:** Qian Zhang, Xin Song, Ping Ma, Liping Lv, Yangyang Zhang, Jiang Deng, Yanyu Zhang

**Affiliations:** ^1^Institute of Health Service and Transfusion Medicine, Academy of Military Medical Sciences, Beijing, China; ^2^Beijing Key Laboratory of Blood Safety and Supply Technologies, Beijing, China; ^3^Department of Otolaryngology Head and Neck Surgery, Chinese People's Liberation Army (PLA) General Hospital, Beijing, China

**Keywords:** cytomegalovirus, viral miRNAs, US33as-5p, IFNAR1, immune evasion

## Abstract

As the first line of antiviral defense, type I interferon (IFN) binds IFN receptor 1 (IFNAR1) and IFNAR2 to activate the Jak-STAT signal transduction pathway, producing IFN-stimulated genes (ISGs) to control viral infection. The mechanisms by which human cytomegalovirus (HCMV) counteracts the IFN pathway are only partially defined. We show that miR-US33as-5p encoded by HCMV is expressed in both lytic and latent infection. By analysis with RNA hybrid and screening with luciferase reporter assays, we identified IFNAR1 as a target of hcmv-miR-US33as-5p, which was further verified by examining the expression of two IFNAR1 mutants and the binding of IFNAR1 to miR-US33as-5p/miR-US33as-5p-M1/miR-US33as-5p-M2. We found that after the transfection of miR-US33as-5p mimics into different cell lines, the phosphorylation of downstream proteins and ISG expression were downregulated. Immunofluorescence showed that the miR-US33as-5p mimics also inhibited STAT1 translocation into the nucleus. Furthermore, we constructed HCMV with mutant miR-US33as-5p and determined that the mutation did not affect HCMV replication. We found that MRC-5/human foreskin fibroblast (HFF) cells infected with ΔmiRNA HCMV exhibited higher IFNAR1 and ISG expression and a reduced viral load in the presence of exogenous IFN than cells infected with WT HCMV did, confirming that the knockout of miR-US33as-5p impaired viral resistance to IFN. Finally, we tested the effect of ΔmiRNA HCMV on THP-1 and d-THP-1 cells, common *in vitro* models of latent infection and reactivation, respectively. Again, we found that cells infected with ΔmiRNA HCMV showed a reduced viral load in the presence of IFN than the control cells did, confirming that miR-US33as-5p also affects IFN resistance during both latency and reactivation. These results indicate a new microRNA (miRNA)-based immune evasion mechanism employed by HCMV to achieve lifelong infection.

## Introduction

Human cytomegalovirus (HCMV), a prevalent human pathogen, is a member of the subfamily of β-herpesviruses, which are enveloped, double-stranded (ds) DNA viruses that maintains persistent latent infection for the duration of the host's lifetime (1). HCMV infection can be asymptomatic in healthy individuals but is the major cause of morbidity and mortality in immunocompromised patients and the leading cause of congenital birth abnormalities (2). Current antiviral drugs (such as ganciclovir) have been proven efficacious to control viral infection, but there are few approaches to thoroughly eliminate the virus (3). Therefore, further investigation to identify the mechanism linking viral persistence and immune evasion remains necessary.

MicroRNAs (miRNAs) are short non-coding RNAs (19–22 nucleotides in length) that post-transcriptionally regulate gene expression, causing the degradation and translational inhibition of target mRNAs by base-pairing with the 3′-untranslated region (3′-UTR) through the RNA-induced silencing complex (RISC) (4, 5). More than 230 viral miRNAs have been discovered, but the target genes of these viral miRNAs have not been extensively studied (6). HCMV has a large genome of 230–250 kb and is currently known to encode 26 miRNAs from 16 precursors. However, several miRNAs not included in the miRBase database (http://www.mirbase.org/) have been discovered. For example, we and other researchers found that miR-US33as-5p is encoded by HCMV and expressed in both lytic and latent infection (7).

In previous studies on herpesviruses, many viral miRNAs were found to be expressed to avoid host immune mechanisms, allowing persistent infection to be maintained, which is particularly common in β- and γ-herpesviruses (8). UL-112-1, miR-BART2-5p, and miR-K12-7, which are encoded by HCMV, Epstein-Barr virus (EBV) and Kaposi's sarcoma-associated herpesvirus (KSHV), respectively, were found to target MHC class I-related chain B (MICB), a stress-induced ligand that is targeted by natural killer (NK) cells through the ligand NKG2D (9, 10). miR-K12-9, encoded by KSHV, reduces NF-κB signaling by binding IRAK1 and MyD88, leading to reduced levels of the inflammatory cytokines IL-6 and IL-8 (11). miR-UL112-1, US5-1, and US5-2, encoded by HCMV, targets multiple cellular targets, reducing the secretion of the inflammatory cytokines TNF-α and IL-6 (12). miR-UL148D-1 targets RANTES, regulating activation, and normal T-cell expression and secretion (13, 14). Although some HCMV miRNAs and their targets have been reported, many more miRNAs, their target antiviral mechanisms, and their biological functions remain unclear.

In this study, we found that hcmv-miR-US33as-5p targets interferon (IFN) receptor 1 (IFNAR1) to evade host immunity by interfering with the canonical IFN signaling pathway. Type I IFNs play a key role in innate immune responses against viral infection through the production and secretion of IFNs by host cells upon the recognition of viral nucleic acids. Moreover, IFNs protect cells from further enhanced viral infection by inducing ISGs, many of which encode antiviral proteins. We showed that overexpression of hcmv-miR-US33as-5p obviously downregulated the expression of IFNAR1, further leading to inhibition of the Jak-STAT signal transduction pathway. Besides, mutation of miR-US33as-5p in HCMV obviously decreased viral resistance to IFN in both lytic and latent infection. These results establish a mechanism of hcmv-miR-US33as-5p-based immune evasion utilized by HCMV.

## Materials and Methods

### Cells and Viruses

MRC-5 cells were cultured in minimum Eagle's medium (MEM, Gibco, Shanghai, China), HEK293 (293) cells and human foreskin fibroblast (HFF) cells were cultured in Dulbecco's modified Eagle's medium (DMEM, Gibco), and THP-1 cells were cultured in RPMI 1640 medium (Gibco). All media were supplemented with 10% fetal bovine serum (FBS), 100 U/mL penicillin, and 100 μg/mL streptomycin, and all cells were cultured at 37°C under a 5% CO_2_ atmosphere. Confluent cell monolayers were starved of serum for 4 h before drug treatment. Cells were treated with 10 μg/mL cycloheximide (CHX, Monmouth Junction, NJ, USA) for 0.5 h, maintained in serum-free DMEM, washed with phosphate-buffered saline (PBS) three times, and then stimulated with 1,500/500 IU/mL IFNα (Sigma-Aldrich, St. Louis, MO, USA) in medium supplemented with 10% FBS until harvesting.

HCMV (Toledo strain) was routinely inoculated and propagated in MRC-5 cells, and aliquots were stored at −80°C. MRC-5 and HFF cells were infected with HCMV at a multiplicity of infection (MOI) of 1, and THP-1 cells were infected with HCMV at an MOI of 10. For HCMV infection, the cells were infected with HCMV in a medium without FBS for 2 h, after which the medium was replaced with a medium containing 5% FBS.

### Plasmid Construction

The 3′-UTRs of hcmv-miR-US33as-5p targets predicted by the RNA hybrid procedure were amplified from mRNA-derived complementary DNA (cDNA) with the corresponding primers listed in Supplementary 1. After purification and digestion, the fragments were cloned into the pmirGLO dual-luciferase vector (Promega, Madison, WI, USA). In addition, vectors encoding IFNAR1 with one of two binding-site mutations were produced as described in Supplementary 2 and named IFNAR1-M1 and IFNAR1-M2 (depicted in **Figure 2B**).

The sequence of hcmv-miR-US33as-5p was similarly amplified with miR-US33as-5p-specific primers, and the fragment was cloned into the GV251 vector (GeneChem, Shanghai, China), with the resulting vector named GV251-miR-US33as-5p. Two vectors encoding the mutants IFNAR1-M1 and IFNAR1-M2 were individually generated and named US33as-M1 and US33as-M2, respectively, as described in Supplementary 3.

To construct a RED-flagged STAT1 expression vector, STAT1 was amplified using semi-nested PCR (outer forward primer: 5′-TGCGTAGCTGCTCCTTTGGT-3′, outer reverse primer: 5′-GTCAGGATCCACTTCAGACACAGAAATCAA-3′; inner forward primer: 5′-GTCAGTCGACATGTCTCAGTGGTACGAACT-3′, inner reverse primer: 5′-GTCAGGATCCACTTCAGACACAGAAATCAA-3′). Fragments were cloned into the pDsRed1-N1 vector (Takara), and the resultant vector was named pDs-RED-STAT1. Insertion of the construct was confirmed by the company AuGCT.

### Dual-Luciferase Reporter Assays

To confirm the target of hcmv-US33as-5p, 293 cells were cultured in 24-well plates. The cells were co-transfected with 150 ng of pmirGLO vector containing the 3′-UTR of a predicted target gene and 350 ng of GV251 blank vector or vector containing US33as-5p/US33as-5p-M1/US33as-5p-M2 using jetPRIME (Polyplus, Illkirch, France) in triplicate wells according to the manufacturer's instructions. The cell medium was replaced after 4 h of transfection. Luciferase activities were determined at 48 h post-transfection (hpi) according to the manufacturer's instructions (Dual-Luciferase Reporter Assay System, Promega) using a luminometer. The transfection efficiency was normalized by the Renilla luciferase activity in the corresponding well.

### Western Blot Analysis

Cell pellets were lysed in RIPA lysis buffer (CWBio, Beijing, China) with a cocktail of protein inhibitors and phosphatase inhibitors (CWBio). Extracts were separated by 10% sodium dodecyl sulfate-polymerase gel electrophoresis (SDS-PAGE), after which proteins were transferred to polyvinylidene difluoride (PVDF) membranes (Sigma-Aldrich) and visualized with antibodies specific for IFNAR1 (A1715), IFNAR2 (A1769), STAT1 (A0027), STAT2 (A14995), and β-actin (A2319) (all from ABclonal, Wuhan, Hubei, China), p-STAT1 (7649T), p-STAT2 (4441T), p-JAK1 (74129S), JAK1 (3344T), p-Tyk2 (68790S), and Tyk2 (14193S) (all from CST, Boston, MA, USA); and HRP-conjugated goat anti-rabbit IgG (ABclonal) via an electrochemiluminescence (ECL) detection system, followed by exposure using a ChemiDoc^TM^ XRS+ imaging system (Bio-Rad, Hercules, CA, USA).

### RT-qPCR

Total RNA was isolated from cells using TRIzol reagent (Sigma-Aldrich), chloroform, and isopropanol according to the manufacturer's protocols. RNA purity and quantity were detected by a UV-2700 spectrophotometer (Shimadzu, Kyoto, Japan). cDNA was reverse transcribed from the extracted RNA using ReverTra Ace qPCR RT Master Mix with gDNA Remover (TOYOBO, Osaka, Japan).

The expression levels of IFNAR1, IFNAR2, and 6 ISGs (primers listed in Supplementary 4) were screened and normalized to GAPDH mRNA expression (forward primer: 5′-TCGCTCTCTGCTCCTCCTGTTC-3′, reverse primer: 5′-CGCCCAATACGACCAAATCC-3′). The expression levels of other genes relative to the control were calculated as fold changes.

### Real-Time PCR

The total viral copy number in cell supernatants of infected cells was determined with a genomic DNA extraction kit (BioTeke, Beijing, China) according to the manufacturer's instructions. Changes in HCMV DNA load were monitored by absolute quantitative real-time PCR with SYBR Green (TOYOBO). HCMV DNA levels were detected using primers specific for the HCMV IE1 gene (forward primer: 5′-ATGTACGGGGGCATCTCTCT-3′, reverse primer: 5′-GGCTTGGTTATCAGAGGCCG-3′).

### mRNA Stability

MRC-5 cells transfected with mimics were treated with 10 μg/10^6^ cells/mL actinomycin D (ActD) at 48 hpi. Samples were taken at 0, 2, 4, 6, and 8 h post-treatment and processed for qPCR analysis. MRC-5 cells treated with ActD at the specific concentration for 8 h did not show signs of overt ActD toxicity.

### Examination of miRNA

miRNA expression was determined with a modified protocol from our previous report (15). Briefly, cDNA synthesis such that a poly(A) tail followed each miRNA was performed by reverse transcription (RT) using a tagged poly(T) primer (5′-CAGGTCCAGTTTTTTTTTTTTTTTVN-3′) and the One-Step miRNA cDNA Synthesis Kit (Hai-gene Bio Inc., Harbin, China). The generated cDNA was then used for miRNA examination (primers listed in Supplementary 5). The relative expression level of each miRNA was normalized to the expression of U6, and the cycle threshold (CT) ranged from 15 to 17 cycles.

### Immunofluorescence

Two hundred and ninety three cells were transfected with GV251 blank vector or vector containing US33as-5p, followed by selection with G418 (0.5 μg/mL) for 3 days. The cells were then transfected with the pDs-RED-STAT1 vector for 24 h and then stimulated with or without IFNα (1,500 IU/mL) for 12 h. STAT1 nuclear localization was detected by indirect immunofluorescence using an Immunol Fluorescence Staining Kit (Beyotime, Beijing, China) according to the manufacturer's instructions. Fluorescence was visualized with an Olympus FluoView 1000 confocal microscope.

### hcmv-33as-5p Knockout With the CRISPR-Cas9 System

hcmv-33as-5p was knocked out with the lentiCRISPR v2 plasmid as described previously (16, 17). Briefly, sgRNAs targeting hcmv-US33as-5p sequences capped by a 5′-N_20_GG PAM sequence was designed using an online tool (https://zlab.bio/guide-design-resources). The sgRNAs were then synthesized and annealed and ligated to the linearized vector. Lentivirus was produced at a high titer in 293 cells by co-transfection with 2.6 μg of CRISPRv2 vector, 0.25 μg of pVSVg plasmid (Addgene 8454), and 2.5 μg psPAX2 plasmid (Addgene 12260). MRC-5 cells were infected with the Cas9/sgRNA lentivirus (MOI = 0.5), and stable transfectants were selected using puromycin (2.5 μg/mL). The MRC-5 cells were then infected with HCMV (Toledo strain, MOI = 1), and the supernatant, which contained the mutant virus, was collected at 72 hpi. The mutant virus was seeded in 96-well plates containing MRC-5 cells for plaque assays and isolated as described previously (18).

### Statistical Analyses

All analyses were performed using SPSS software. All data are shown as the means ± standard deviation (SD). Significant differences (*p* < 0.05) were determined by one-way analysis of variance (ANOVA) followed by Dunnett's *t*-test or Kruskal-Wallis test followed by the Mann-Whitney U test. All experiments in this study were performed at least twice.

## Results

### IFNAR1 Is a Putative Target of hcmv-miR-US33as-5p

**Figure 1:**
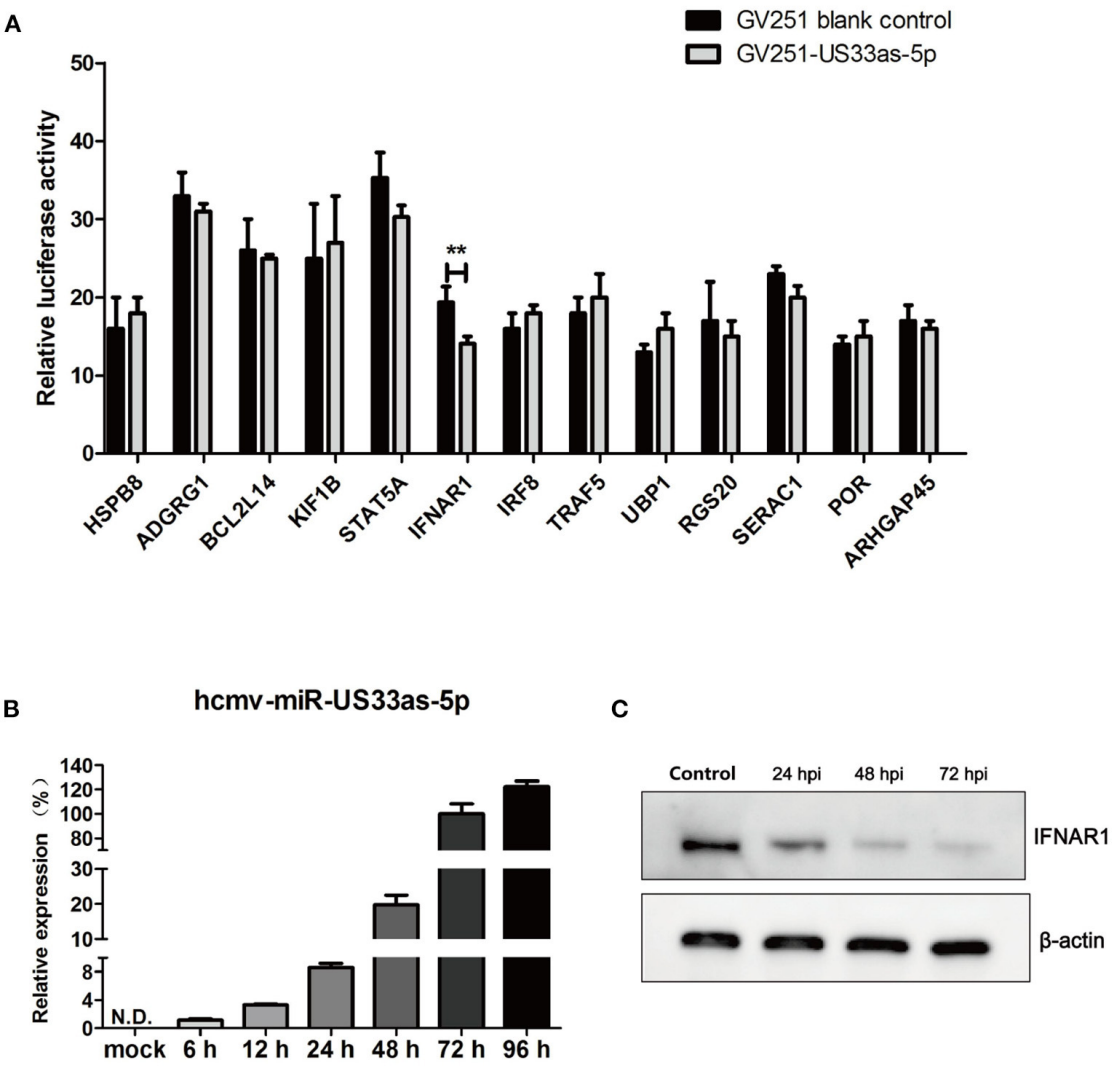
hcmv-miR-US33as-5p inhibited the expression of IFNAR1 by targeting the 3′-UTR of IFNAR1. **(A)** Reporter vectors containing the 3′-UTRs of 13 predicted target genes were co-transfected with GV251 blank vector or vector containing US33as-5p into 293 cells. The dual-luciferase reporter assay was used to determine the relative Renilla luciferase activity normalized to firefly luciferase activity in cells in the corresponding wells at 48 h post-transfection (hpi) when cell lysates were harvested. **(B)** The expression of hcmv-US33as-5p during HCMV infection at different time points was determined by qPCR. U6 was used as a loading control. **(C)** IFNAR1 protein expression at different time points after the transfection of US33as-5p mimics was examined by western blot analysis. The sample of the control group comes from cells transfected with NC-RNA for 72 h. β-Actin was used as a loading control. The assays were performed in triplicate wells, and data were collected from two different experiments and are represented as the means ± SDs; **p < 0.05.

To investigate the influence of hcmv-miR-US33as-5p on viral infection, 13 putative target mRNAs and their hcmv-miR-US33as-5p-binding sites were identified using RNAhybrid (http://bibiserv.techfak.uni-bielefeld.de/rnahybrid/). Detailed information on the targets is provided in Table 1, and all putative targets were selected from the human genome database. To further validate these miRNA targets, we constructed 13 pmirGLO-reporter plasmids with primers analyzed by BLAST (Supplementary 1). To determine potential targets, the ability of hcmv-miR-US33as-5p to bind the 3′-UTR of each putative mRNA was verified by dual-luciferase reporter assay. From the 13 predictive targets, two targets were confirmed, and the relative luciferase activity in cells co-transfected with pmirGLO-STAT5A-UTR or pmirGLO-IFNAR1-UTR and hcmv-miR-US33as-5p mimics was obviously downregulated by 14.2% (*p* = 0.072) and 27.3% (*p* < 0.05), respectively, compared with that in cells co-transfected with negative control miRNA (Figure 1A). Since STAT5 is a transcription factor involved in IFN signaling, we concentrated on the IFNAR1-encoded protein, which activates Jak-STAT signaling and functions as an antiviral factor.

**Table 1:** Putative hcmv-miR-US33as-5p targets identified by RNA hybrid.

**Putative target mRNA**	**Accession number**	**Position of binding site**	**Mfe**
Homo sapiens TNF receptor associated factor 5 (TRAF5)	NM_145759	3′UTR	−28.2 kcal/mol
Homo sapiens interferon alpha and beta receptor subunit 1 (IFNAR1)	NM_000629	3′UTR	−30.9 kcal/mol
Homo sapiens signal transducer and activator of transcription 5A (STAT5A)	NM_003152	3′UTR	−35.7 kcal/mol
Homo sapiens adhesion G protein-coupled receptor G1 (ADGRG1)	NM_201524	5′UTR	−33.0 kcal/mol
Homo sapiens serine active site containing 1 (SERAC1)	NM_032861	3′UTR	−29.3 kcal/mol
Homo sapiens kinesin family member 1B (KIF1B)	NM_015074	3′UTR	−35.7 kcal/mol
Homo sapiens upstream binding protein 1 (UBP1)	NM_014517	3′UTR	−32.1 kcal/mol
Homo sapiens BCL2 like 14 (BCL2L14)	NM_030766	3′UTR	−29.2 kcal/mol
Homo sapiens regulator of G protein signaling 20 (RGS20)	NM_170587	3′UTR	−30.5 kcal/mol
Homo sapiens cytochrome p450 oxidoreductase (POR)	NM_000941	3′UTR	−30.9 kcal/mol
Homo sapiens Rho GTPase activating protein 45 (ARHGAP45)	NM_012292	3′UTR	−29.7 kcal/mol
Homo sapiens interferon regulatory factor 8 (IRF8)	NM_002163	3′UTR	−27.9 kcal/mol
Homo sapiens heat shock protein family B (small) member 8 (HSPB8)	NM_014365	3′UTR	−29.1 kcal/mol

We next investigated the expression of hcmv-miR-US33as-5p during HCMV replication. MRC-5 cells were infected with HCMV (Toledo strain), and total RNA and protein were collected at different time points following infection. Based on melt curve analysis, the relative expression levels were calculated as fold changes relative to the level at 72 hpi. The expression of hcmv-miR-US33as-5p was increased with HCMV replication (Figure 1B). In addition, 293 cells were transfected with hcmv-miR-US33as-5p mimics/negative control (NC)-RNA (100 nM) and incubated with 1,500 U/mL IFNα The expression of INFAR1 was determined by western blot analysis. The results showed that the expression of IFNAR1 was reduced at different times postinfection (Figure 1C). These results indicate that hcmv-miR-US33as-5p may target IFNAR1 by binding the 3′-UTR of IFNAR1 mRNA.

### Mutation of IFNAR1-Binding Sites Abolished the Targeting of IFNAR1 by hcmv-miR-US33as-5p, Which Was Restored by Back Mutation of the hcmv-miR-US33as-5p Seed Region

**Figure 2:**
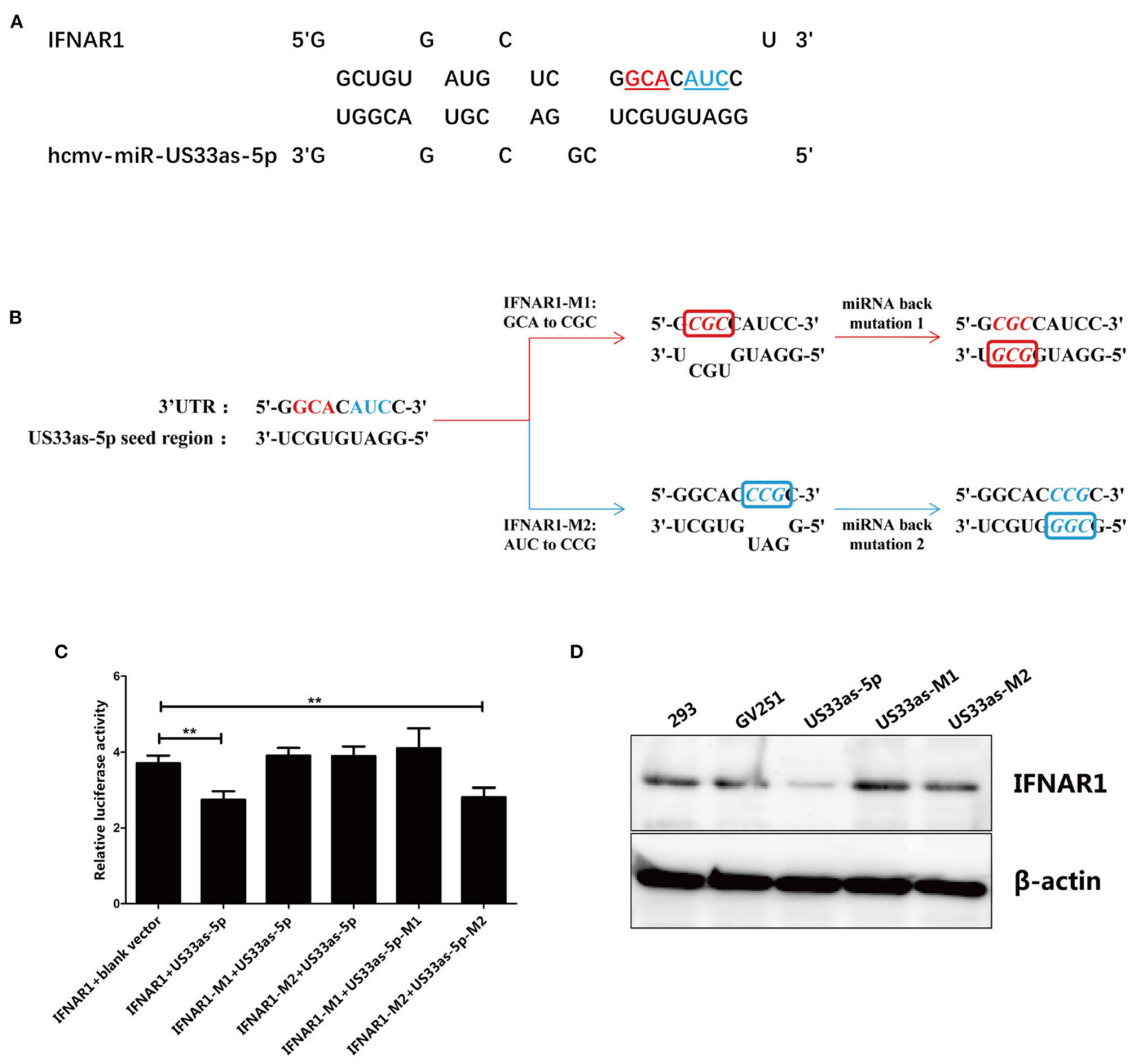
Main binding site in hcmv-miR-US33as-5p for IFNAR1 mRNA. **(A)** Schematic of the targeting of IFNAR1 by hcmv-miR-US33as-5p. Mutated nucleotides are shown in color and underlined. **(B)** Mutation of the IFNAR1-binding site and back mutation of the hcmv-miR-US33as-5p seed region. For IFNAR1 mutagenesis 1, GCA was mutated to CGC, and the miRNA was complementarily mutated to GCG for miR-US33as-5p back mutagenesis 1. For IFNAR1 mutagenesis 2, AUC was mutated to CCG, and the miRNA was complementarily mutated to CGG for miR-US33as-5p back mutagenesis 2. The pmirGLO-IFNAR1-Mutation-UTR vector and GV251-US33as-Mutation-UTR vector were co-transfected into 293 cells cultured in 24-well plates. **(C)** Dual-luciferase reporter assays to determine relative firefly luciferase activity in 293 cells co-transfected with pmirGLO-IFNAR1-Mutation-UTR vector and GV251-US33as-Mutation-UTR vector were performed, and the calculated data are shown in Figure 1A. **(D)** Western blot showing the expression of IFNAR1 in 293 cells co-transfected with pmirGLO-IFNAR1(-Mutation)-UTR vector and GV251-US33as(-Mutation)-UTR vector harvested at the same time, with β-actin used as a loading control. The assays were performed in triplicate wells, and data were collected from two different experiments and are represented as the means ± SDs; **p < 0.05.

The putative IFNAR1-binding sites in hcmv-miR-US33as-5p identified by RNAhybrid are listed in Figure 2A. To confirm the exact binding site in hcmv-miR-US33as-5p for IFNAR1, we designed two mutations in the IFNAR1-binding site. To generate the first mutant, the nucleotides GCA were mutated to CGC, and to generate the second, the nucleotides AUC were mutated to CCG; these sequences were cloned into the pmirGLO vector, and the resultant plasmids were named IFNAR1-M1 and IFNAR1-M2, respectively. The complementary nucleotide sequences of the hcmv-miR-US33as-5p seed region corresponding to the mutated sites in IFNAR1 were mutated into GCG (US33as-M1) or CGG (US33as-M2) and then cloned into the GV251 vector. The mutagenesis and back mutagenesis protocols are explicitly described in Figure 2B.

The pmirGLO-IFNAR1-Mutation-UTR vectors (for the expression of WT IFNAR1, IFNAR1-M1, or IFNAR1-M2) and GV251-US33as-Mutation-UTR vectors (for the expression of blank vector, US33as, US33as-M1, and US33as-M2) were co-transfected into 293 cells cultured in 24-well plates. Dual-luciferase reporter assays were used to determine the fluorescence intensity. The grouping and results are listed in Figure 2C. Compared with 293 cells co-transfected with IFNAR1+blank vector, 293 cells co-transfected with IFNAR1+US33as showed significantly decreased relative luciferase activity, which was then found to be significantly increased in IFNAR1-M1/IFNAR1-M2+US33as-co-transfected 293 cells. These results indicate that base pairing between the seed region of US33as-5p and the 3′-UTR of IFNAR1 (regardless of the nucleotides at the M1 or M2 site) plays a key role in miRNA function. This is consistent with the findings that mutation of IFNAR1-binding sites disturbed hcmv-miR-US33as-5p binding to IFNAR1 and that back mutation of the hcmv-miR-US33as-5p seed region restored this binding ability, which was validated by western blot analysis (Figure 2D). Expression of the IFNAR1 protein was clearly reduced in GV251-US33as-UTR-transfected 293 cells compared to negative control- or GV251-transfected 293 cells, but this reduction was abolished by GV251-US33as-Mutation-UTR transfection.

Interestingly, although base pairing between both IFNAR1-M1+US33as-M1 and IFNAR1-M2+US33as-M2 was correct, the relative luciferase activity upon the transfection of IFNAR1-M1+US33as-M1 did not differ from that of the control group, while relative luciferase activity upon the transfection of IFNAR1-M2+US33as-M2 showed a significant difference compared with that of the control group, indicating that the base sequence at the M1 site plays a more important role in miRNA function than that at the M2 site.

### hcmv-miR-US33as-5p Blocks the Jak-STAT Signaling Transduction Pathway by Targeting IFNAR1

Jak-STAT signaling is a necessary cytokine receptor signaling pathway that is important in many processes, including growth regulation, survival, differentiation, and especially pathogen clearance. Blockade of the binding of type I IFNs to their cell surface receptor (IFNAR1) reduces a series of signaling events in the target cells, therefore undoubtedly inhibiting the conserved Jak-STAT pathway. Notably, HCMV has been demonstrated to encode several factors that counteract the IFN signaling pathway, including HCMV tegument protein pp65 (19–22) and pp71 (23); therefore, a subsequent series of experiments were performed by the transfection of miRNA mimics without HCMV infection, with IFNAR2 also examined as a control target.

**Figure 3:**
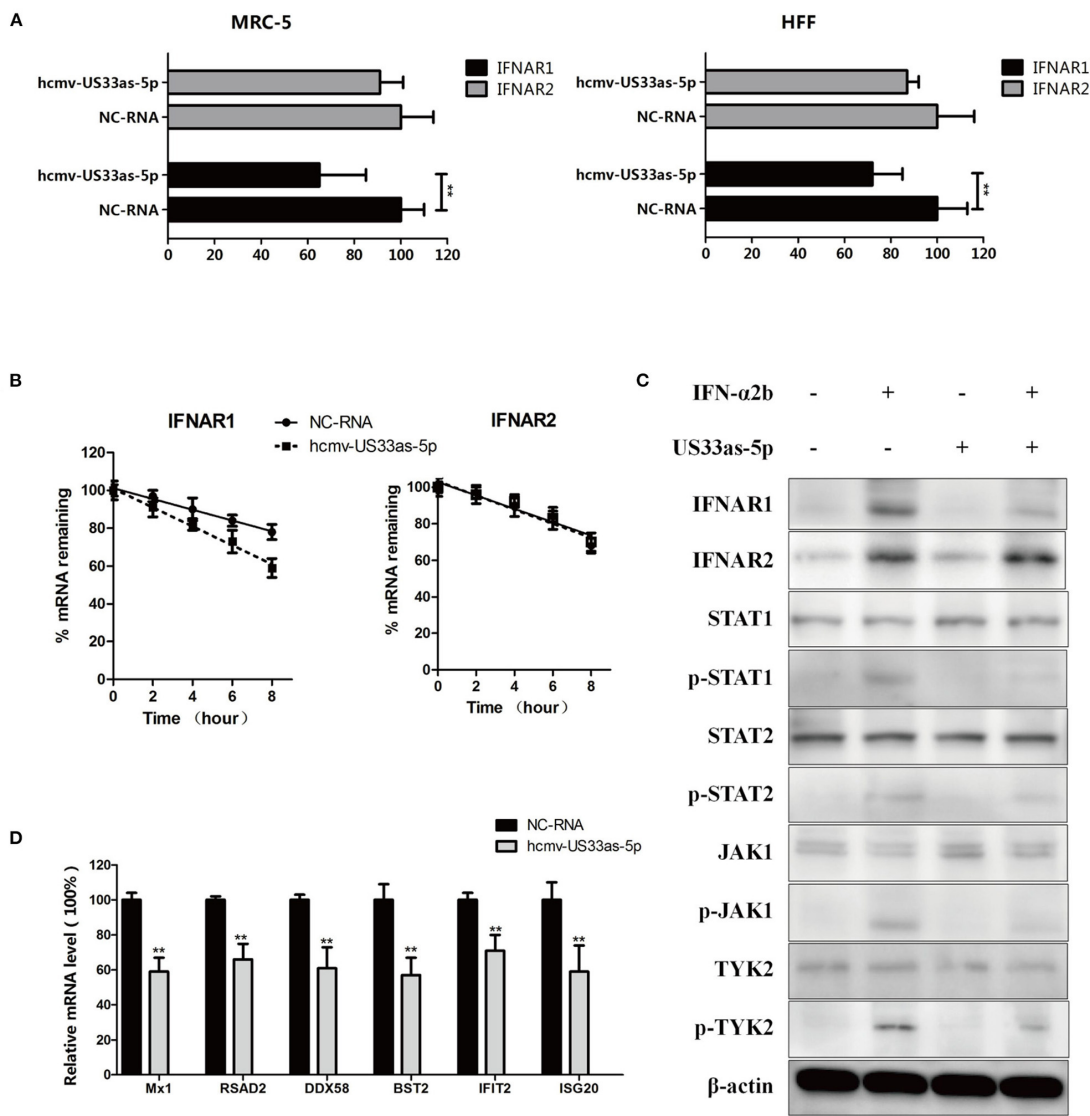
hcmv-miR-US33as-5p downregulates IFNAR1 and Jak-STAT, limiting the release of ISGs. **(A)** MRC-5 and HFF cells were cultured in 24-well plates and transiently transfected with hcmv-US33as-5p mimics or a negative control RNA (NC-RNA) at a concentration of 100 nM for 24 h. The cells were treated with 10 μg/mL CHX, followed by stimulation by 1,500 U/mL IFNα for 1 h until harvest. The expression of IFNAR1 and IFNAR2 was determined by qPCR. **(B)** The mRNA stability of IFNAR1 and IFNAR2 in MRC-5 cells transfected with US33as-5p mimics or NC-RNA and incubated with actinomycin D to inhibit transcription is presented as the amount of mRNA detected at a given time relative to that at 0 h, which was set as 100%. **(C)** Immunoblotting of lysates from MRC-5 cells underwent treatment similar to that described in Figure 3A and prolonged incubation with 1,500 U/mL IFN-α for 6 h. was performed. Blots were probed for IFNAR1, IFNAR2, STAT1, STAT2, Jak1, Tyk2 and their phosphorylated forms. β-Actin was run as a loading control. **(D)** The expression of multiple ISGs (Mx1, RSAD2, DDX58, BST2, IFIT2, and ISG20) in MRC-5 cells was determined by qPCR. The RNA harvested from MRC-5 cells underwent treatment similar to that described in Figure 3C. GAPDH was amplified as a loading control. The assays were performed in triplicate wells, and data were collected from two different experiments and are represented as the means ± SDs; **p < 0.05.

Both MRC-5 and HFF cells were cultured in 24-well plates and transiently transfected with hcmv-US33as-5p mimics or NC-RNA at a concentration of 100 nM for 24 h, followed by 12 h co-culture of serum-free mediums. The cells were then treated with 10 μg/mL CHX to inhibit the synthesis of endogenous IFN, followed by stimulation with 1,500 U/mL IFNα for 1 h until harvest. The mRNA levels were examined by qPCR, and the results showed that both MRC-5 and HFF cells had significantly lower INFAR1 mRNA expression than NC-transfected cells. In contrast, the expression of IFNAR2, which does not have a binding site for miRNA, was not affected by hcmv-miR-US33as-5p mimics (Figure 3A). To investigate this further, we treated the transfected MRC-5 cells with ActD, a transcription inhibitor. The cells transfected with hcmv-miR-US33as-5p mimics showed a rapid decline in IFNAR1 expression but no difference in IFNAR2 expression (Figure 3B). Taken together, these results suggest that the hcmv-miR-US33as-5p mimics obviously reduced IFNAR1 expression.

Prolonged IFN stimulation for 6 h was carried out to further investigate changes in the downstream pathway. We next evaluated differences in the expression of IFNAR1 and its downstream proteins, which are key proteins in the Jak-STAT signaling pathway, by western blot analysis. The cells transfected with hcmv-miR-US33as-5p showed lower levels of IFNAR1, phosphorylated STAT1, STAT2, Jak1 and Tyk2, suggesting decreased activation of the Jak-STAT signaling pathway, while IFNAR2 showed no difference among groups (Figure 3C). In addition, the ISGs Mx1, RSAD2, DDX58, BST2, IFIT2, and ISG20 were selected to analyze changes in ISG mRNA levels by qRT-PCR. The mRNA expression of these ISGs in cells transfected with hcmv-miR-US33as-5p mimics was lower than that in cells transfected with NC-RNA (Figure 3D). Therefore, these results showed that hcmv-miR-US33as-5p inhibits the Jak-STAT signaling pathway.

**Figure 4:**
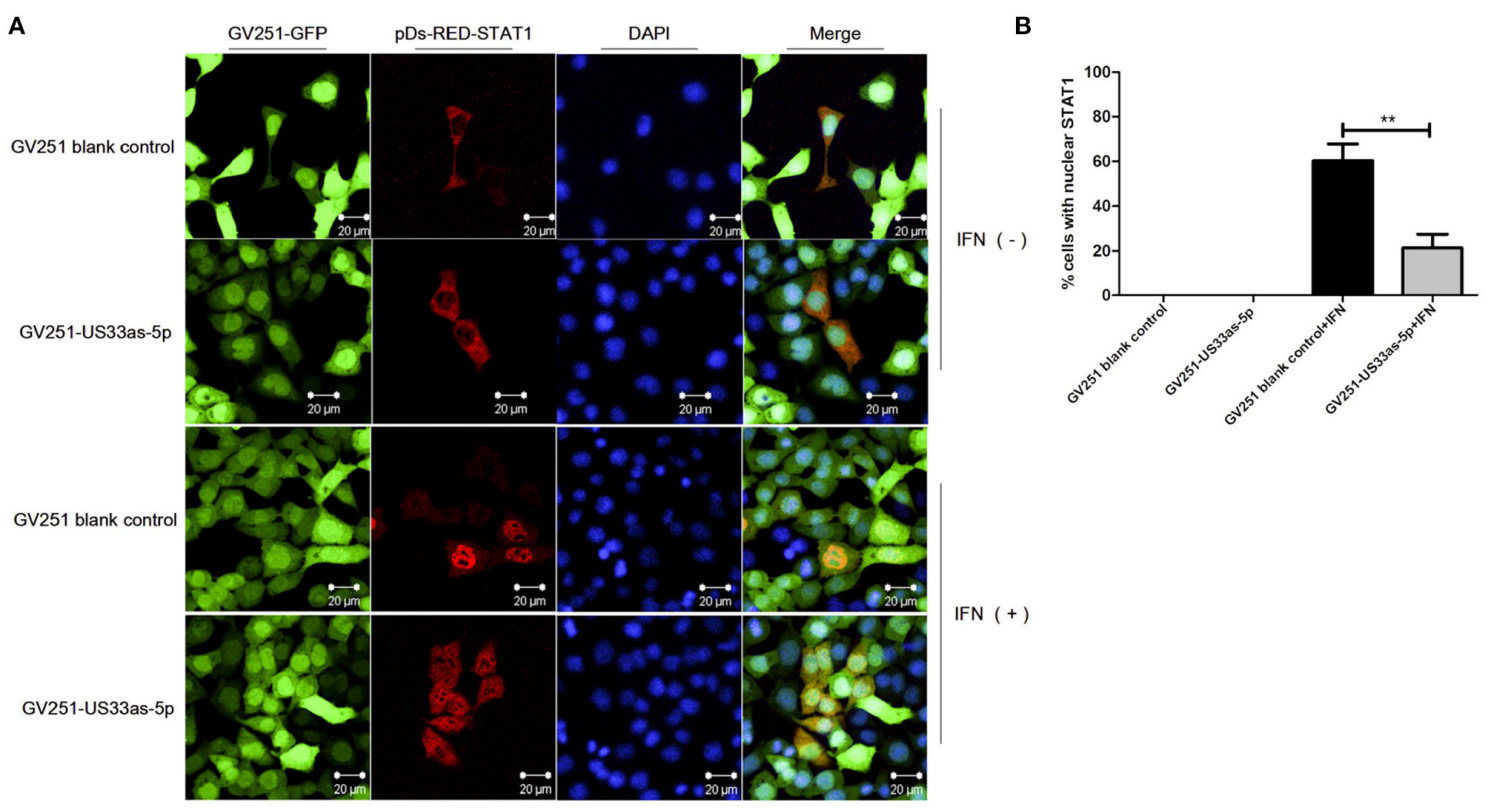
hcmv-miR-US33as-5p inhibits the nuclear translocation of STAT1. **(A)** 293 cells were transfected with GV251 blank vector or vector containing US33as-5p, followed by selection with G418 (0.5 μg/mL) for 3 days. The cells were then transfected with the pDs-RED-STAT1 vector for 24 h and then stimulated with or without IFNα (1,500 IU/mL) for 12 h. STAT1 nuclear localization was detected by indirect immunofluorescence. **(B)** Percentage of cells with nuclear STAT1 from experiments performed in A. Cells were counted in three random fields and the results are expressed as mean ± SD (**p < 0.01). Fluorescence was visualized with an Olympus FluoView 1000 confocal microscope. All images were captured under a 60× objective lens.

Moreover, immunofluorescence was performed to determine whether hcmv-miR-US33as-5p could inhibit the nuclear translocation of STAT1. As Figures 4A,B shows, without IFNα treatment, STAT1 was retained in the cytoplasm in both GV251 blank- and GV251-US33as-5p-transfected cells. After stimulation with IFNα, STAT1 translocated into the nucleus in the GV251 blank-transfected control group but was still retained in the cytoplasm of GV251-US33as-5p-transfected cells. All of the above results reveal that hcmv-miR-US33as-5p can downregulate IFNAR1, STAT1 and ISGs and inhibit STAT1 nuclear translocation by targeting IFNAR1.

### HCMV in Which hcmv-miR-US33as-5p Was Knocked Out Was Produced and Verified

To further investigate the effect of hcmv-miR-US33as-5p on viral infection, HCMV with hcmv-miR-US33as-5p knockout was established by the CRISPR-cas9 system. A lentivirus CRISPR-cas9 system targeting HCMV miR-US33as-5p was designed by constructing Cas9- and sgRNA-co-expressing lentiviruses. After recombined lentiviral infection and selection with puromycin, the CRISPR-cas9 system was constructed in MRC-5 cells. These cells were then infected with WT HCMV, and mutant viruses in the supernatant were harvested at 72 hpi. After further plaque assays and the isolation of viral mutants three times, the pure ΔmiRNA HCMV strain was obtained, amplified, and stocked.

**Figure 5:**
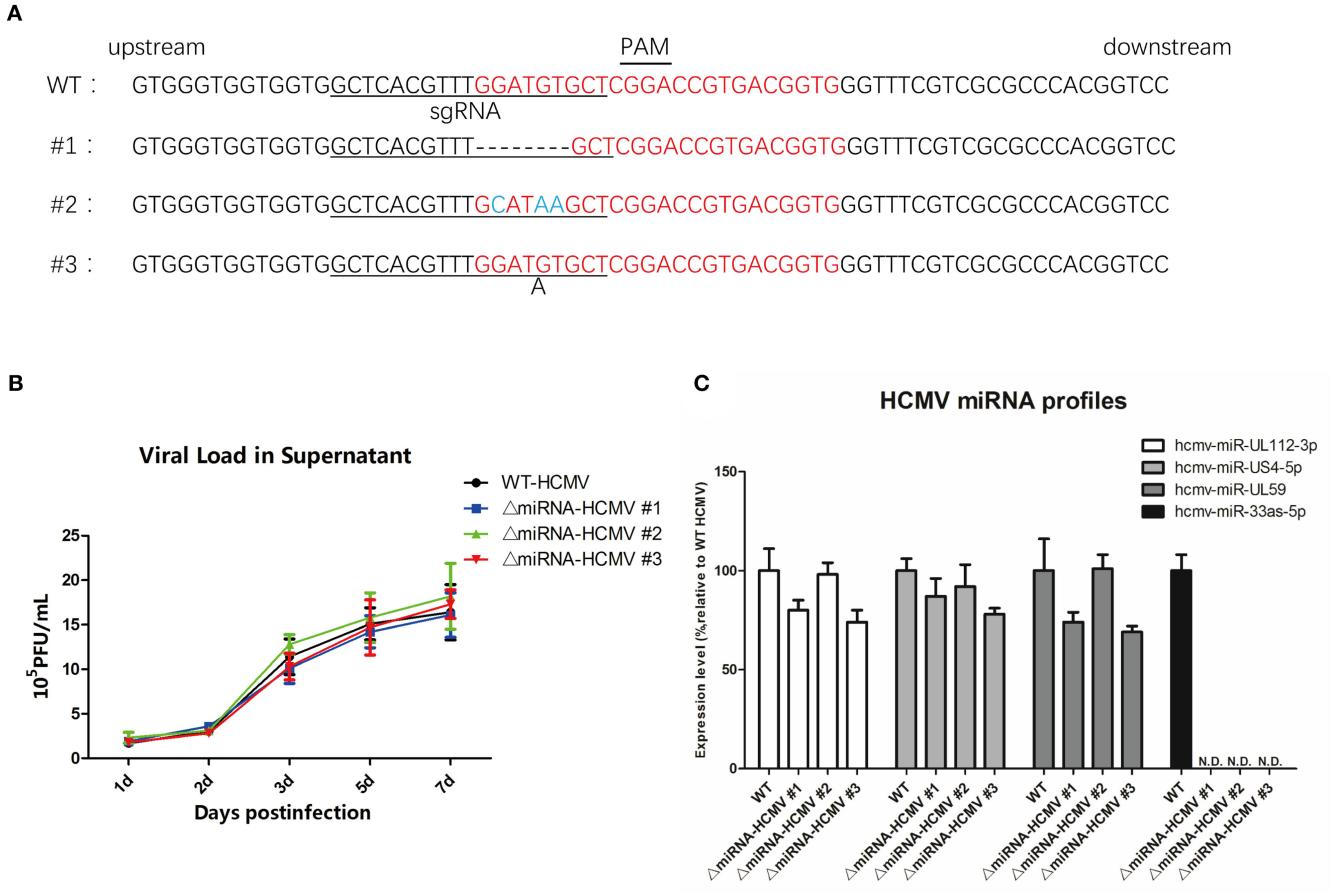
CRISPR-Cas9 introduced mutations in hcmv-US33as-5p and did not obviously affect HCMV virology. **(A)** The DNA sequence surrounding the hcmv-US33as-5p editing region from each HCMV mutant strain was amplified by PCR and analyzed by DNA sequencing. The PAM sequence and sgRNA-targeting region are indicated with lines. The DNA sequence-editing miRNA bases are highlighted in red. Deletions and insertions are presented by dashed lines or a letter underneath the sequence, respectively. No mutations in the control samples were observed. **(B)** MRC-5 cells were infected with WT, ΔmiRNA HCMV #1, ΔmiRNA HCMV #2, or ΔmiRNA HCMV #3 at an MOI = 1, and the viral loads from the supernatants at 1d, 2d, 3d, 5d, 7d were determined by qPCR. **(C)** The expression of several miRNAs encoded by HCMV at 72 hpi was examined by qPCR.

To determine whether the targeting region was mutated, DNA was extracted from the three mutant strains of HCMV and Sanger sequenced (Figure 5A, forward primer: 5′-AGCGGTCGTGCTTGTCTTTA-3′; reverse primer: 5′-ACGTGGTCCGTCGAAATTGA-3′). We observed that the CRISPR-cas9 system-induced indel mutations in the regions surrounding hcmv-miR-US33as-5p. To further evaluate whether genome editing affected viral proliferation and other miRNAs, MRC-5 cells were separately infected with mutated and WT HCMV, and the viral load was determined at different times (Figure 5B). The results indicate that genome editing did not impair viral replication. In addition, analysis of several viral miRNAs at 72 hpi showed no differences in the expression of these other viral miRNAs (Figure 5C). HCMV mutant strain #1 (named ΔmiRNA HCMV) was selected and investigated in the next series of experiments.

### Mutation of hcmv-miR-US33as-5p Impaired Viral Resistance to IFN

**Figure 6:**
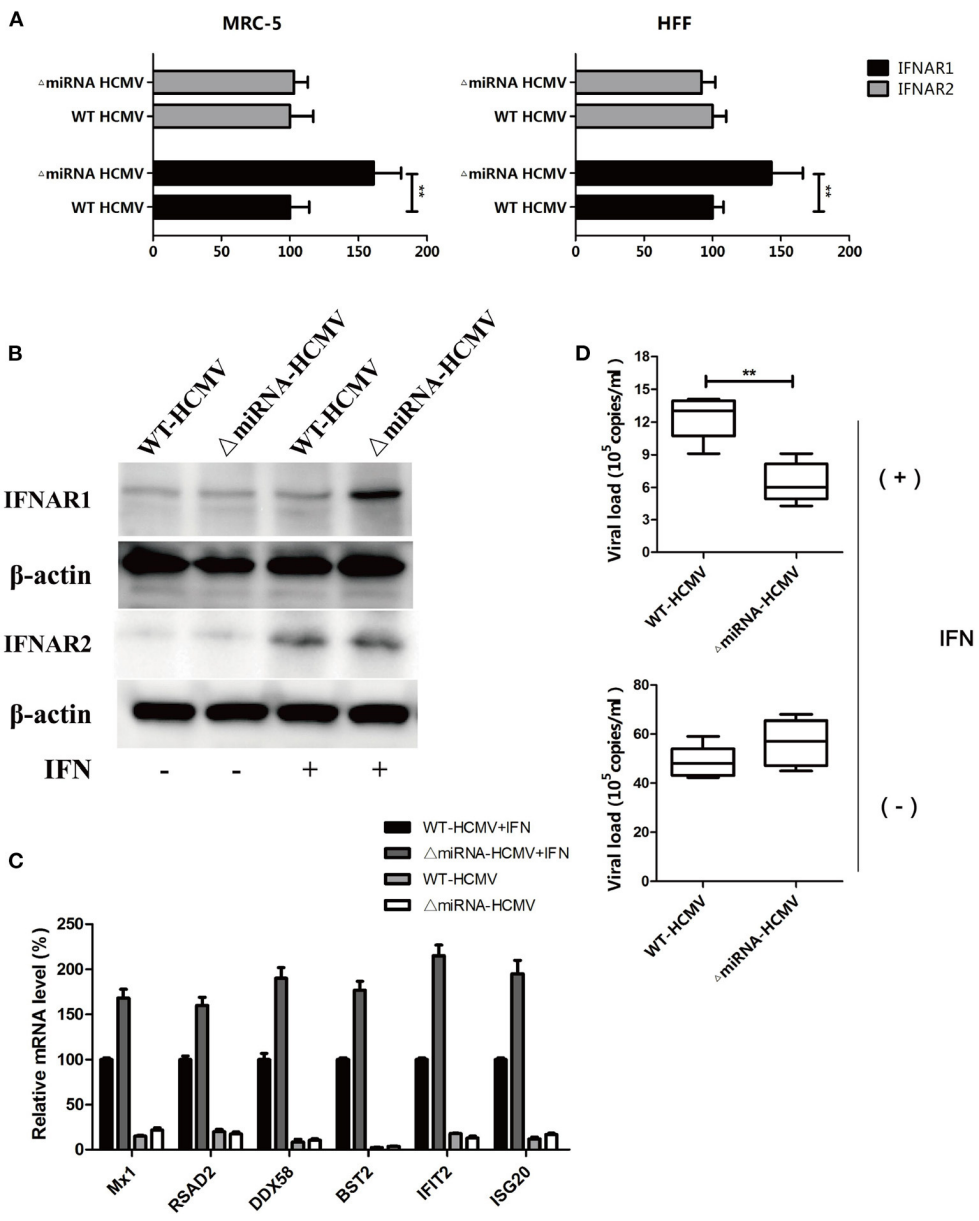
Knockout of hcmv-US33as-5p alleviated blockade of the IFN pathway. **(A,B)** MRC-5 and HFF cells were infected with WT or ΔmiRNA HCMV for 48 h, followed by treatment with CHX and stimulation with IFN for 24 h. The expression of IFNAR1 and IFNAR2 was determined by qPCR and western blot. **(C)** The expression of multiple ISGs was examined by qPCR. **(D)** Total DNA was isolated from the supernatants, and HCMV copy numbers were determined and calculated by qPCR. **p < 0.05.

Given the effect of genome editing on the targeting region, we next investigated whether mutation would affect viral infection. Both MRC-5 and HFF cells were infected with WT or ΔmiRNA HCMV for 48 h, followed by treatment with CHX and stimulation with IFN for 24 h. The RNA and DNA were then collected for further analysis. The transcription of IFNAR1 was obviously higher in cells infected with ΔmiRNA HCMV. However, there was no difference in the transcription of IFNAR2 (Figure 6A). The results were furtherly determined by western blot (Figure 6B). In addition, the relative mRNA levels of all 6 selected ISGs in the WT HCMV-infected cells were obviously lower than those in the ΔmiRNA HCMV-infected cells in the presentation of IFN stimulation (Figure 6C). Furthermore, the HCMV copy number in ΔmiRNA HCMV-infected MRC-5 cells was significantly lower than that in the WT HCMV-infected MRC-5 cells in the presentation of IFN, while no obvious difference was seen between groups without IFN incubation (Figure 6D). These results indicate that inhibition of hcmv-miR-US33as-5p enhanced the expression of ISGs and weakened HCMV replication, which could impair the resistance of HCMV to IFN.

### hcmv-miR-US33as-5p Is Essential for Latent Viral Infection and Viral Reactivation

Many researchers agree with the view that the differentiation of monocytes into macrophages *in vivo* may represent a pivotal process triggering the recurrence of the latent virion, furtherly rendering CMV to disseminate into host tissues (24–27). Among the *in vitro* cell lines to mimic the natural infection, human monocytic leukemia cells (THP-1) and differentiated THP-1 cells (d-THP-1) are universal models in which HCMV latency and reactivation are investigated (6, 28, 29). Considering that herpesvirus miRNAs play key roles in latency and reactivation, the biological function of hcmv-miR-US33as-5p was investigated in THP-1 and d-THP-1 cells.

**Figure 7:**
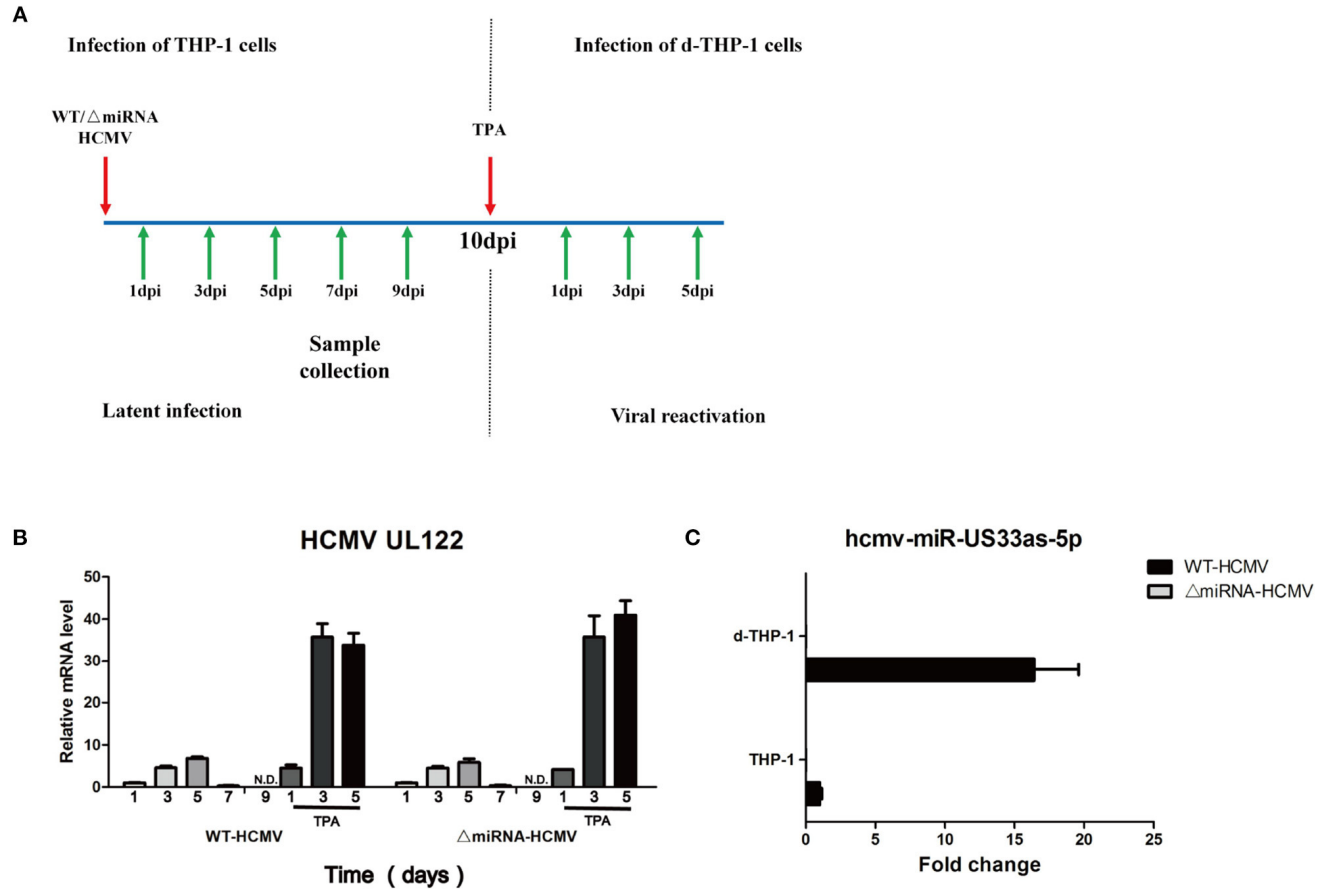
hcmv-US33as-5p is expressed in both viral latency and reactivation. **(A)** A schematic diagram depicting *in vitro* models of latent infection and viral reactivation. THP-1 cells were infected with WT or ΔmiRNA HCMV (MOI = 10) and maintained for 10 days in the culture medium. Then, the THP-1 cells were differentiated into macrophages (d-THP-1) by stimulated with TPA in order to render the cells permissive to HCMV infection and further maintained in the medium for another 5 days. **(B)** The expression of viral UL122 at 1, 3, 5, 7, and 9 dpi and 1, 3, and 5 days after TPA stimulation was detected by qPCR. **(C)** The expression of hcmv-US33as-5p at 9 dpi and 3 days after TPA stimulation was determined by qPCR.

THP-1 cells were infected with WT or ΔmiRNA HCMV and maintained for 10 days in culture medium. Then, the THP-1 cells were stimulated with TPA at 50 ng/mL and further maintained in medium for another 5 days (Figure 7A). Expression of the IE gene (UL122) was not detected until 9 days post-infection (dpi) in THP-1 cells and detected after 1 day of TPA induction in d-THP-1 cells with both WT and ΔmiRNA HCMV infection, indicating that the latency and reactivation models had been successfully established (Figure 7B). We next investigated the expression of hcmv-miR-US33as-5p at different stages of infection (Figure 7C). Samples were collected from cells at 9 dpi and 3 days after TPA induction, representative of viral latency and reactivation, respectively. As expected, the expression of hcmv-miR-US33as-5p was not detected in cells infected with ΔmiRNA HCMV. In addition, analysis of miRNA expression indicated that hcmv-miR-US33as-5p was expressed both in latency and reactivation. However, its expression exhibit a higher level in reactivation than in latency. This result suggests that miRNAs can affect viral infection in both latency and reactivation.

**Figure 8:**
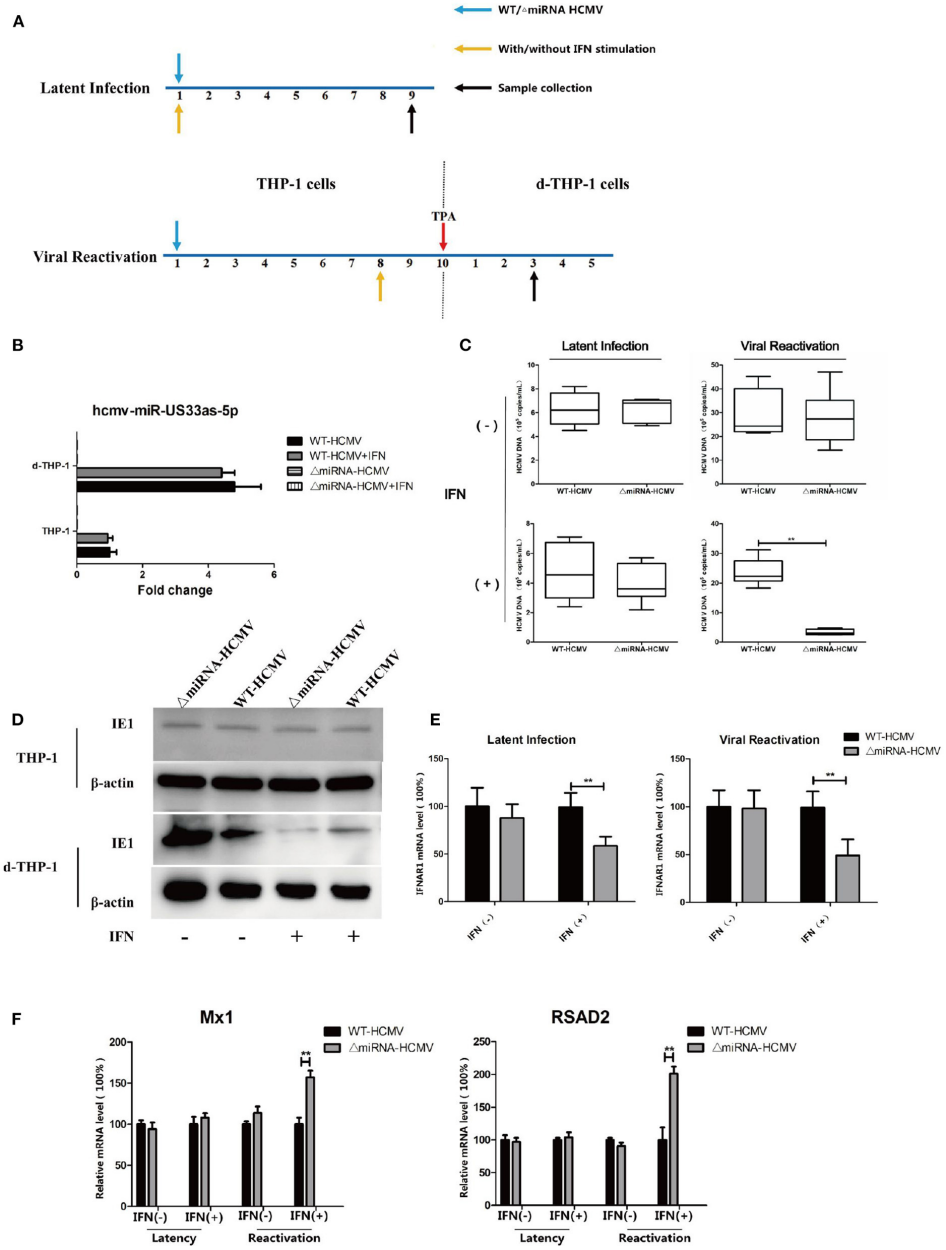
A lack of hcmv-US33as-5p impaired viral resistance to the IFN pathway during latency and reactivation. **(A)** A schematic diagram depicting the *in vitro* models of latent infection and viral reactivation. For latent infection, THP-1 cells were infected with WT or ΔmiRNA HCMV (MOI = 10) and maintained for 9 days in a culture medium supplemented with or without IFN (500 IU/mL). For viral reactivation, THP-1 cells were infected and maintained for 10 days in the culture medium. Then, the THP-1 cells were stimulated with TPA and further maintained in the medium for another 5 days. The cells were stimulated in a culture medium supplemented with or without IFN (500 IU/mL) beginning at 8 dpi. **(B)** The expression of miRNA among groups was examined by qPCR.THP-1 **(C,D)** The viral loads at indicated days were determined by qPCR and western blot. **(E,F)** The expression of IFNAR1 and ISGs (Mx1, RSAD2) among groups were determined by qPCR. **p < 0.05.

In another set of experiments based on the same *in vitro* models of latency and reactivation, THP-1 cells were infected with WT or ΔmiRNA HCMV and stimulated in a culture medium supplemented with or without IFN (500 IU/mL) from infection. The THP-1 cells were further stimulated with TPA to induce differentiation at 10 dpi in viral reactivation groups (see Figure 8A for details). The expression of hcmv-miR-US33as-5p was determined by qPCR (Figure 8B). The viral loads at 9 dpi or 3 days after TPA induction was then determined by qPCR. The results showed no differences in the effects of WT and ΔmiRNA HCMV during latency and reactivation without IFN stimulation and no difference in viral load between the two groups during quiescent infection in the presence of IFN. However, IFN obviously inhibited viral replication in cells infected with ΔmiRNA HCMV during reactivation compared with cells infected with WT HCMV (Figure 8C). The results were furtherly verified by western blot targeting viral IE proteins (Figure 8D). To confirm that hcmv-miR-US33as-5p plays a role in HCMV virology during latency, the mRNA level of IFNAR1 9 dpi in THP-1/d-THP-1 cells was examined (Figure 8E). In cells with IFN stimulation, cells in both latency and reactivation group infected with ΔmiRNA HCMV had obviously higher IFNAR1 expression levels than those infected with WT HCMV, indicating the biological function of miRNAs during quiescent infection and viral recurrence. This is furtherly determined by examination of transcription of downstream ISGs by qPCR (Figure 8F). Taken together, these results suggest that hcmv-miR-US33as-5p serves as a powerful mediator of viral resistance to IFN in latency.

## Discussion

**Figure 9:**
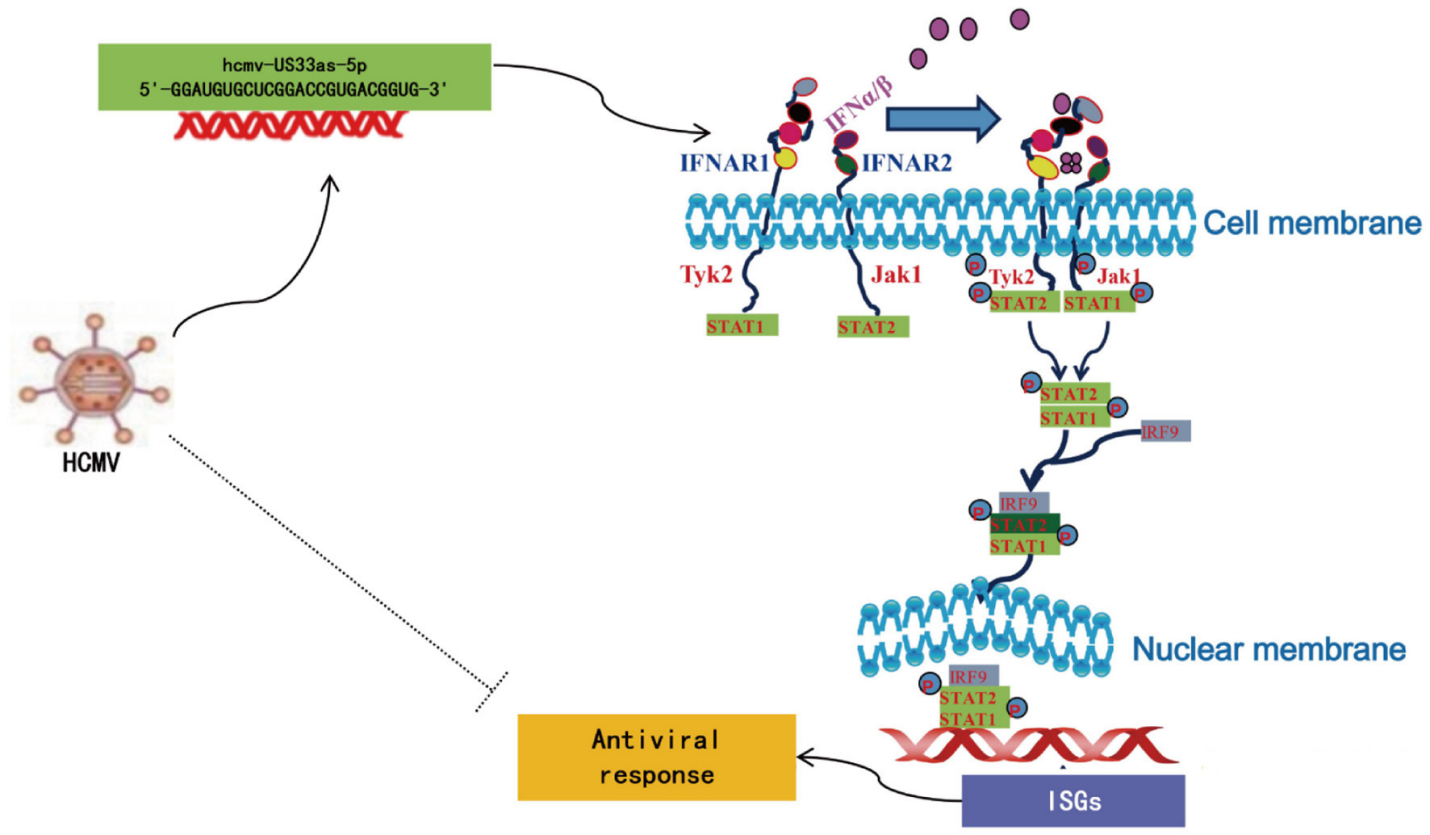
Schematic of the mechanism by which hcmv-US33as-5p facilitates the IFN pathway.

Although the detailed mechanism remains to be fully elucidated, researchers have reported several strategies by which viruses subvert the IFN pathway, such as mechanisms involving pp65, pp71 (pUL82), US9, and IE86, all of which are proteins encoded by HCMV (19, 21–23, 30–36). To the best of our knowledge, this study is the first to report how HCMV miRNA affects the IFN signaling pathway. In this study, we found that hcmv-miR-US33as-5p encoded by HCMV is expressed during both lytic and latent infection. It binds the 3′-UTR of IFNAR1, blocks IFN stimulation, and further inactivates the JAK-STAT signaling pathway, resulting in the limited release of ISGs (Figure 9). HCMV with mutant miR-US33as-5p exhibited a lower resistance to IFN treatment than HCMV with WT miR-US33as-5p. Therefore, we have provided a new approach by which HCMV manipulates the expression of miRNAs to achieve immune evasion.

A total of 26 miRNAs encoded by HCMV are listed in the miRBase database (37), and many of these miRNAs target both viral and cellular genes involved in HCMV processes, such as the maintenance of latency and immune evasion (38, 39). Several miRNAs, such as miR-UL148D and miR-US22, have been reported to play a key role in latency as well as reactivation (13, 40–44). hcmv-miR-US33as-5p, the miRNA discussed in this article, is not included in the miRbase database; however, we and other investigators found that this miRNA is expressed during both lytic and especially latent infection (7). Notably, previous studies analyzed several HCMV-encoded miRNAs expressed during latent infection by directly screening 20 HCMV miRNAs, including miR-UL112-5p, miR-UL36-5p, and miR-UL22A-5p, in human peripheral blood mononuclear cells (PBMCs). However, this screen did not include hcmv-miR-US33as-5p, which maybe because it is not listed in the miRBase database (43).

By analysis with RNAhybrid, we found that hcmv-miR-US33as-5p may interact with the IFN signaling pathway, an approach by which it serves as a pivotal mediator of broad antiviral activity. In the next series of experiments, we found that hcmv-miR-US33as-5p can interact with the 3′-UTR of IFNAR1, especially by base-pairing with its seed region, leading to a reduction in the expression of IFNAR1. Interestingly, we found that the sequence of the M1 site in the miRNA plays a more important role in the biological function of this miRNA than the M2 site, which is near the 5′ end of the miRNA. The results from Figure 3B showed lower levels of transcripts, furtherly indicate that the hcmv-miR-US33as-5p target IFNAR1 by binding the 3′-UTR of IFNAR1 mRNA to inhibit transcription. Because HCMV can employ multiple strategies to defend against the IFN pathway (45), hcmv-miR-US33as-5p mimics in the absence of HCMV were used to investigate changes in downstream signaling pathways. Consistent with our previous hypothesis, hcmv-miR-US33as-5p mimics obviously inhibited the expression of IFNAR1, inhibiting the phosphorylation of STAT1, STAT2, Tyk2, and Jak1, further reducing the expression of downstream ISGs.

IFNs are virus-inducible cytokines that comprise a primordial and tightly regulated defense system against acute viral infection by activating the conserved Jak-STAT signal transduction pathway, which enhances antiviral function and induces immunoregulatory activities (46). IFNs bind the corresponding receptor IFNAR1, stimulating the release of hundreds of ISGs, but only a few of these ISGs have been characterized with respect to their role in antiviral activity (47). For example, the Mx1 gene encodes a guanosine triphosphate (GTP)-metabolizing protein that participates in the cellular antiviral response, antagonizing the replication process in RNA or DNA viruses (48). The protein encoded by the DDX58 gene contains RNA helicase-DEAD box protein motifs and a caspase recruitment domain (CARD) involved in viral dsRNA recognition and regulation of the immune response (49). RSAD2 restricts the replication of a wide range of viruses by modulating cellular metabolic pathways essential for viral replication and/or cell proliferation and survival (50). BST2 inhibits viral replication by tethering enveloped virions to the cell surface to restrict viral release and by inducing the NF-κB-dependent antiviral immune response (51). IFITM2 and other proteins in the IFITM family prevent viruses from traversing the lipid bilayer of the cell and accessing the cytoplasm (52). IFIT2 is involved in a nonspecific antiviral program through its direct interaction with eIF3, which subsequently suppresses translation by more than 60% in cells and viruses during protein synthesis (53). HCMV invasion induces the cellular secretion of IFNs, but this strategy is far less effective than the strategy by which HCMV-encoded miR-US33as-5p targets IFNAR1, which abolishes primary immunomodulatory activity and reduces the release of ISGs. Interestingly, several recent research reported that the association between Covid-19, CMV and inflammageing which potentially leads to higher rates of Covid-19-related mortality (54–56). As miR-US33as-5p would also be active during latent infection, this may become relevant for patients who will experience an IFN response and reactivate CMV. Besides, the impact on IFN and downregulation of IFNAR1 has also been studied in cancer models (57–62). Considering that CMV found to be present in a majority of tumor cells in several forms such as brain tumors, breast, colon, prostate, ovarian cancer as well as in metastases (63–67), it is reasonable to propose that CMV miRNAs are involved in cancer progression as well as IFN treatment, by which further investigation to characterize the role of IFN in tumor invasion is required.

To investigate whether hcmv-miR-US33as-5p plays an important role in real infection, we constructed an HCMV strain in which hcmv-miR-US33as-5p was mutated by CRISPR-Cas9 technology. This genome-editing approach is a convenient way to investigate the role of specific genes in viral activity and was reported in several recent studies (18, 68, 69). We provide evidence that this genome-editing strategy did not have an obvious effect on viral proliferation or the expression of other viral miRNAs. Based on this finding, we tested viral resistance to IFN in MRC-5 and HFF cells, THP-1 cells and d-THP-1 cells, which acted as models of primary infection, latency and reactivation, respectively. Upon the viral infection of MRC-5 and HFF cells, we observed that the expression of ISGs in cells infected with the mutant virus was higher than that in cells infected with the WT virus, and the viral load in cells infected with mutant HCMV was obviously lower than that in cells infected with WT virus, suggesting that hcmv-miR-US33as-5p knockout significantly impaired viral resistance to IFN treatment. These effects were slightly different from those on latency and reactivation. IFN treatment showed an obvious inhibitory effect against viral reactivation; however, IFN treatment failed to reduce latent HCMV infection in THP-1 cells but did alter the expression of IFNAR1. Several factors may contribute to these results: (1) Several other mechanisms may have been employed by HCMV to interact with the type I IFN pathway (30, 31, 33, 35, 36), (2) the IFN incubation period may have been too short for IFN to exhibit inhibitory effects, and (3) a lower viral load during latency may only partially reflect the antiviral effects of IFN treatment. We favor the first hypothesis. Nevertheless, the data revealed that hcmv-miR-US33as-5p is involved in evading host immune attack during latency. A previous study indicated that miR-US4-1 encoded by HCMV in human plasma serves as a biomarker for predicting the efficacy of IFN-α treatment in chronic hepatitis B patients (70). Considering the high prevalence of HCMV in the population, further investigation to reveal the clinical link between HCMV miRNAs and the type I IFN response in remains necessary.

When HCMV enters latency, the expression of major viral proteins is silenced, and the replication cycle of the virus stops. It is believed that viral proteins expressed during latency are recognized by the host immune system, while as a non-immunogenic approach, viral miRNAs benefit viral persistence in the presentation of immune surveillance. Recently, two groups separately identified miR-UL-148D and miR-US22 as playing a key role in viral reactivation, demonstrating that mutant cytomegalovirus (CMV) failed to reactivate in an *in vitro* hematopoietic progenitor cell (HPC) model. Therefore, viral-encoded miRNAs may be ideal in the environment of latent infection, in which cells harbor viruses for long periods and subtle effects on gene regulation may be effective.

In conclusion, our research provides the first details in understanding the molecule mechanisms by which HCMV-encoded miRNAs dampen type I IFN to achieve immune evasion. However, the present study has some limitations. The latency model established in THP-1 cells may be restricted to mimicking quiescent HCMV infection in the host, and more investigations in other experimental HCMV latency models, such as Kasumi-3 cells and CD34+ primary HPCs (43), as well as clinical samples are needed. Therefore, further research to illustrate the role of hcmv-miR-US33as-5p in an *in vivo* model is required and may provide insight into how CMV establishes lifelong infection in the face of the host immune response.

## Data Availability Statement

The datasets presented in this study can be found in online repositories. The names of the repository/repositories and accession number(s) can be found in the article/Supplementary Material.

## Author Contributions

QZ designed, performed, analyzed the *in vitro* experiments, and wrote the manuscript. XS designed and performed an analysis of samples. PM and LL assisted with the design of laboratory methods. YangZ designed and performed statistical analyses. JD and YanyZ designed and oversaw the project and wrote the manuscript. All authors contributed to the article and approved the submitted version.

## Conflict of Interest

The authors declare that the research was conducted in the absence of any commercial or financial relationships that could be construed as a potential conflict of interest.
