# Supplementary material for: Human Cytomegalovirus miR-US33as-5p Targets IFNAR1 to Achieve Immune Evasion During Both Lytic and Latent Infection
Source: Front Immunol. 2021 Mar 5;12:628364. doi: 10.3389/fimmu.2021.628364 (PMC7973039; doi:10.3389/fimmu.2021.628364)

**Supplementary materials and methods**

S-1 Primers of putative targets used to construct pmirGLO-UTR vectors


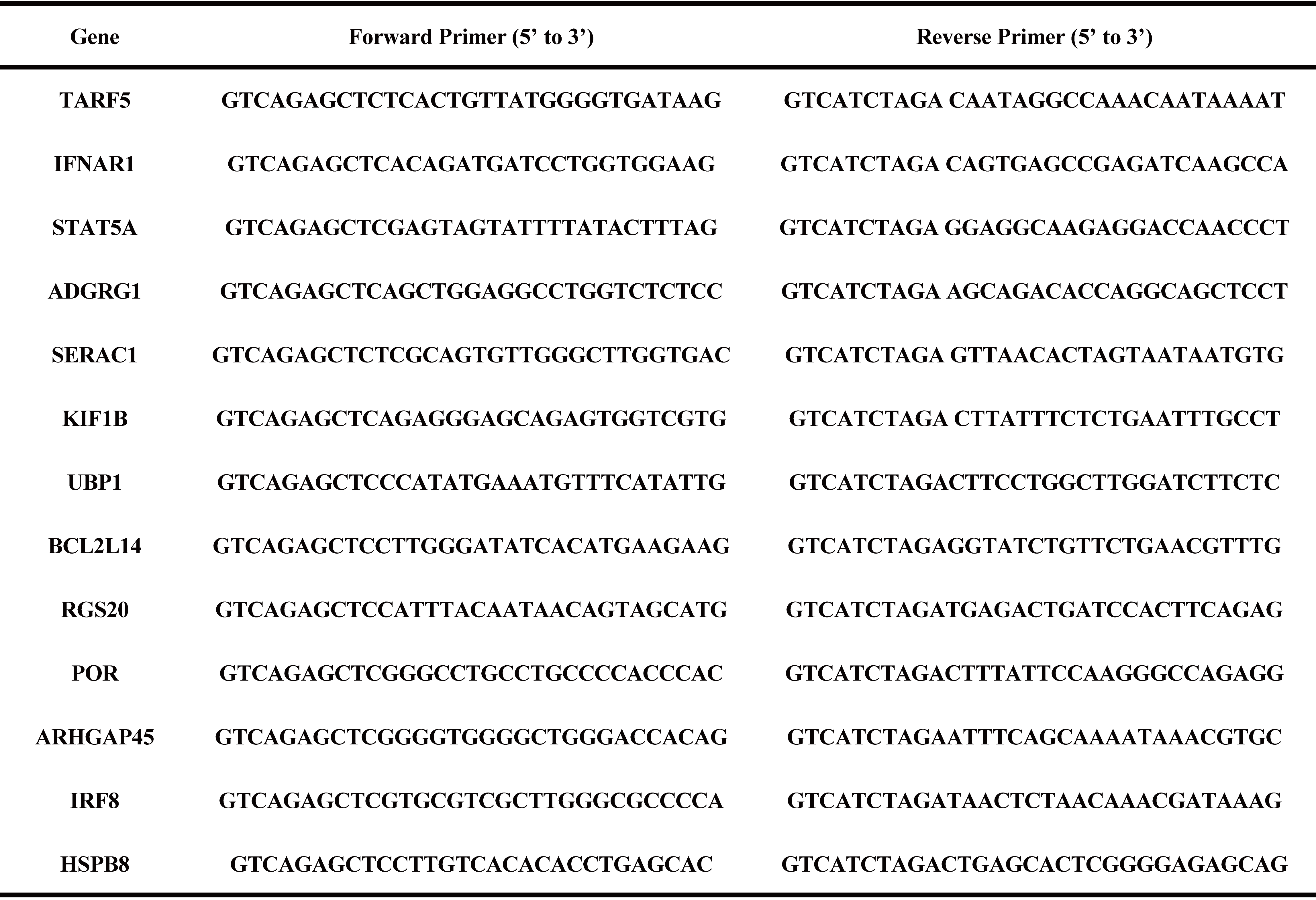


S-2 Construction of vectors containing IFRAR1-M1/IFRAR1-M2

Vectors containing mutant IFRAR1 were established by two rounds of PCR as depicted below. For the first round of PCR, the protocol consisted of 94°C for 3 min for initial denaturation, followed by 25 cycles of 94°C for 30 s, 55°C for 30 s, and 68°C for 30 s for amplification and 68°C for 7 min for final extension.

The second round of PCR consisted of 94 ℃ for 3 min, 50 ℃ for 5 min, and 68 ℃ for 7 min to generate original mutated products, after which the following protocol was used: 94℃ for 3 min for initial denaturation, followed by 30 cycles of 94 ℃ for 30 s, 55 ℃ for 30 s, and 68 ℃ for 30 s for amplification and 68 ℃ for 7 min for final extension.

The final products were purified by agarose gel electrophoresis (TRANSGEN, Beijing, China) and inserted into the pmirGLO firefly luciferase reporter vector (Promega, Madison, WI, USA) according to the manufacturer’s instructions.


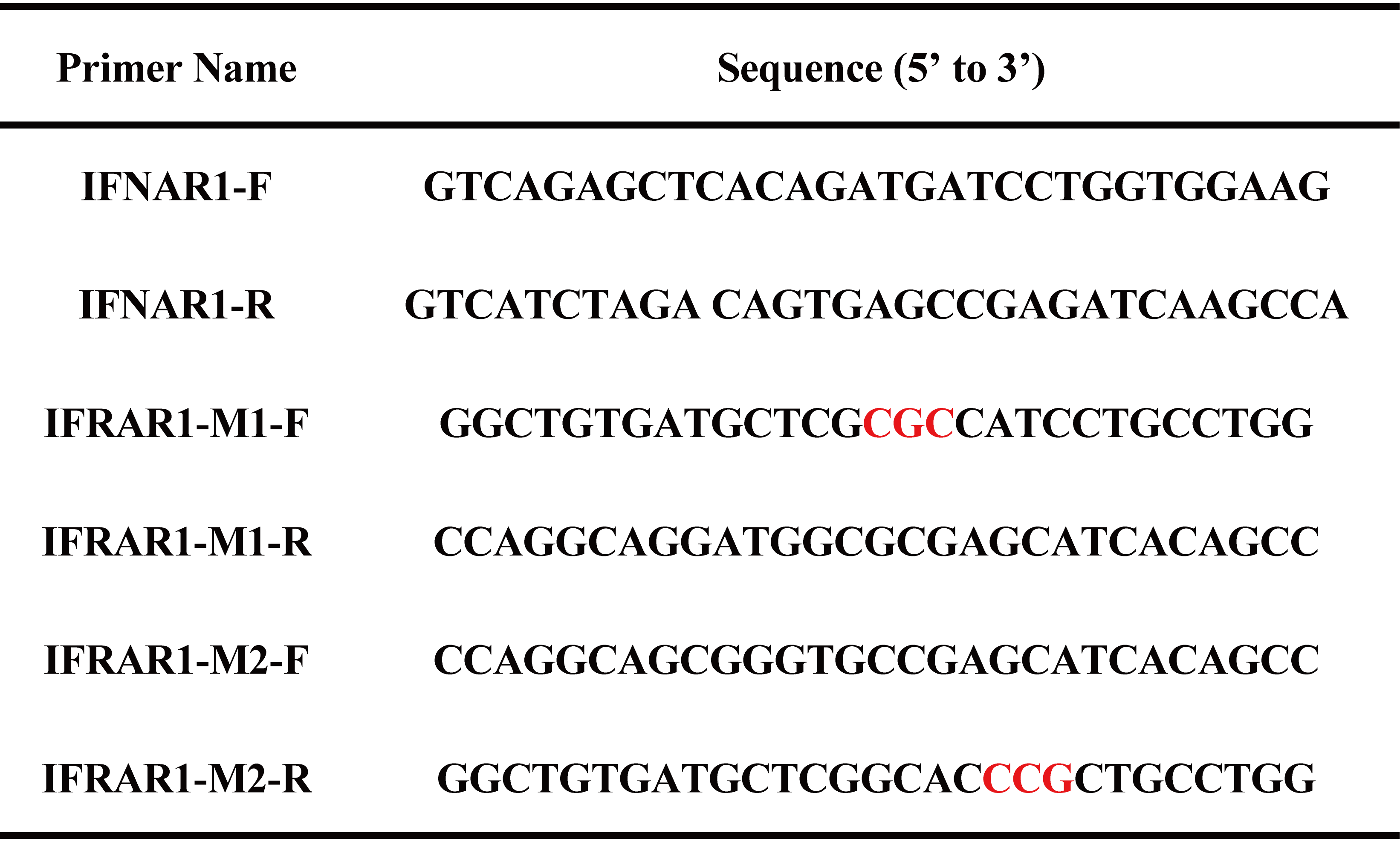


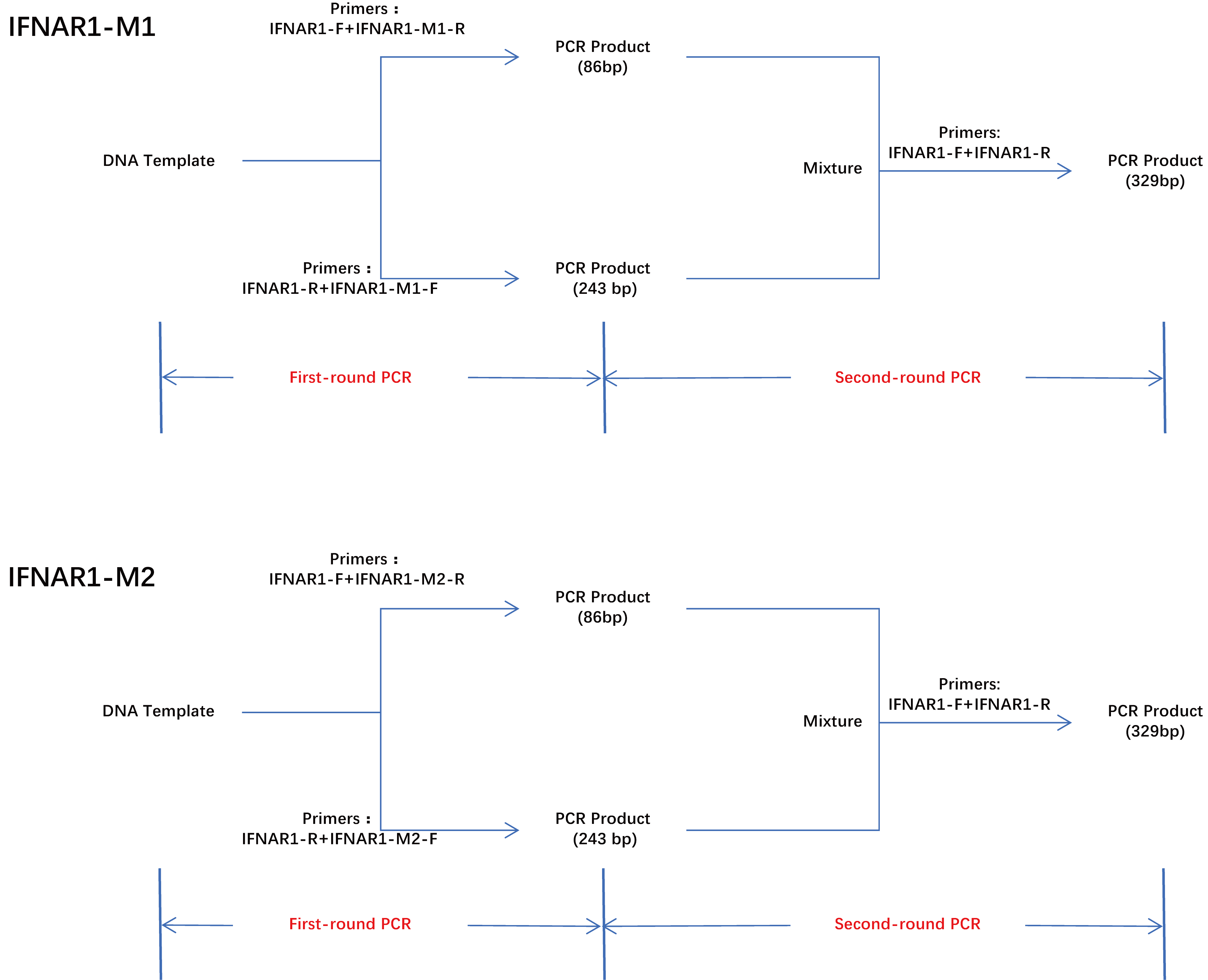


S-3 Primers used to construct the GV251 vector

Mutant vectors were established by two rounds of PCR as depicted below. The following protocol was used for the first round of PCR: 94°C for 3 min for initial denaturation, followed by 25 cycles of 94°C for 30 s, 55°C for 30 s, and 68°C for 30 s for amplification and 68°C for 7 min for final extension.

The second round PCR consisted of 94 ℃ for 3 min, 50 ℃ for 5 min, and 68 ℃ for 7 min to generate original mutant products, followed by 94℃ for 3 min for initial denaturation; 30 cycles of 94 ℃ for 30 s, 55 ℃ for 30 s, and 68 ℃ for 30 s for amplification; and 68 ℃ for 7 min for final extension.

The final products were purified by agarose gel electrophoresis (TRANSGEN, Beijing, China) and inserted into the GV251 vector (GENECHEM, Shanghai, China) according to the manufacturer’s instructions.


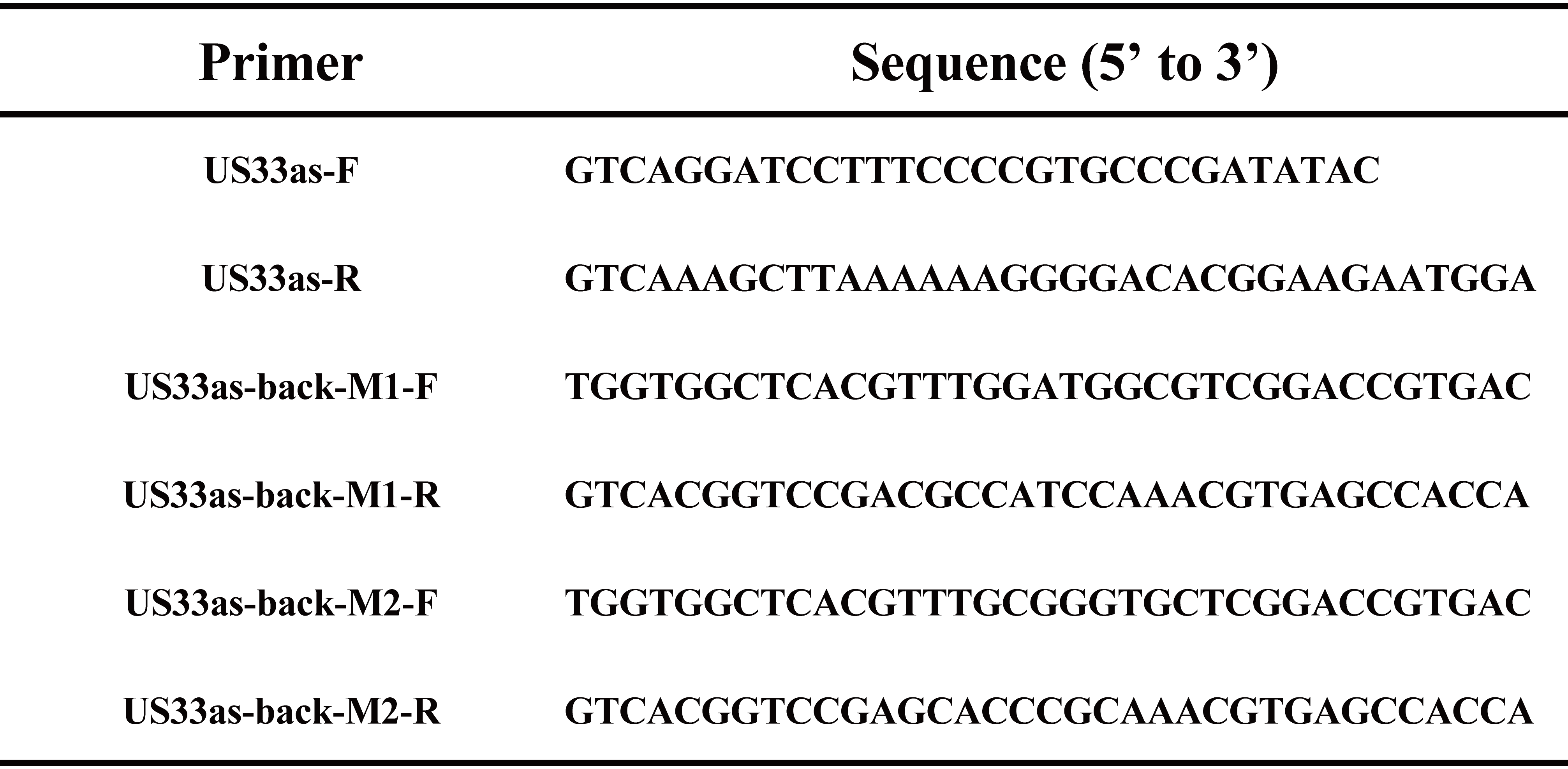


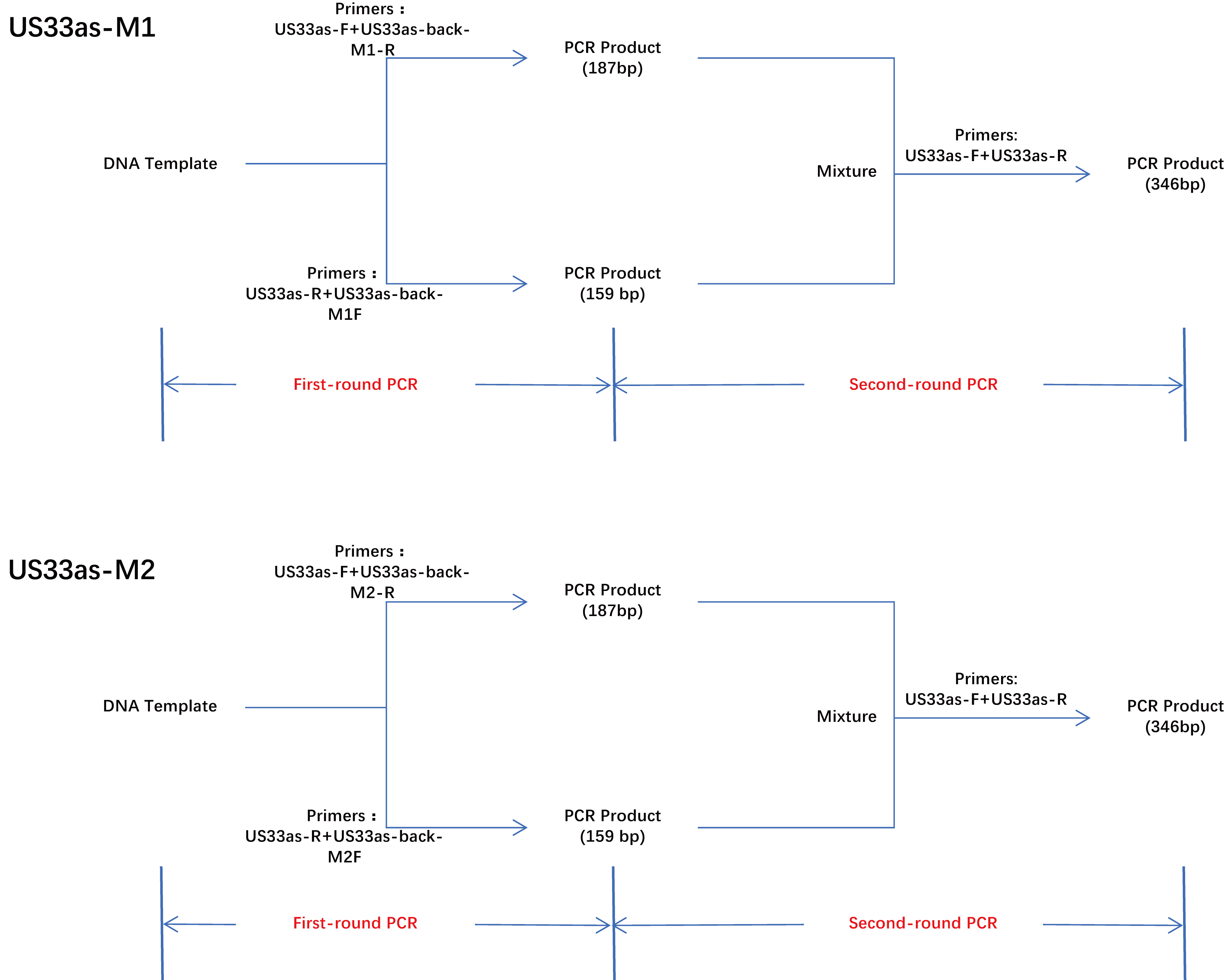


S-4 Primers used for qRT-PCR


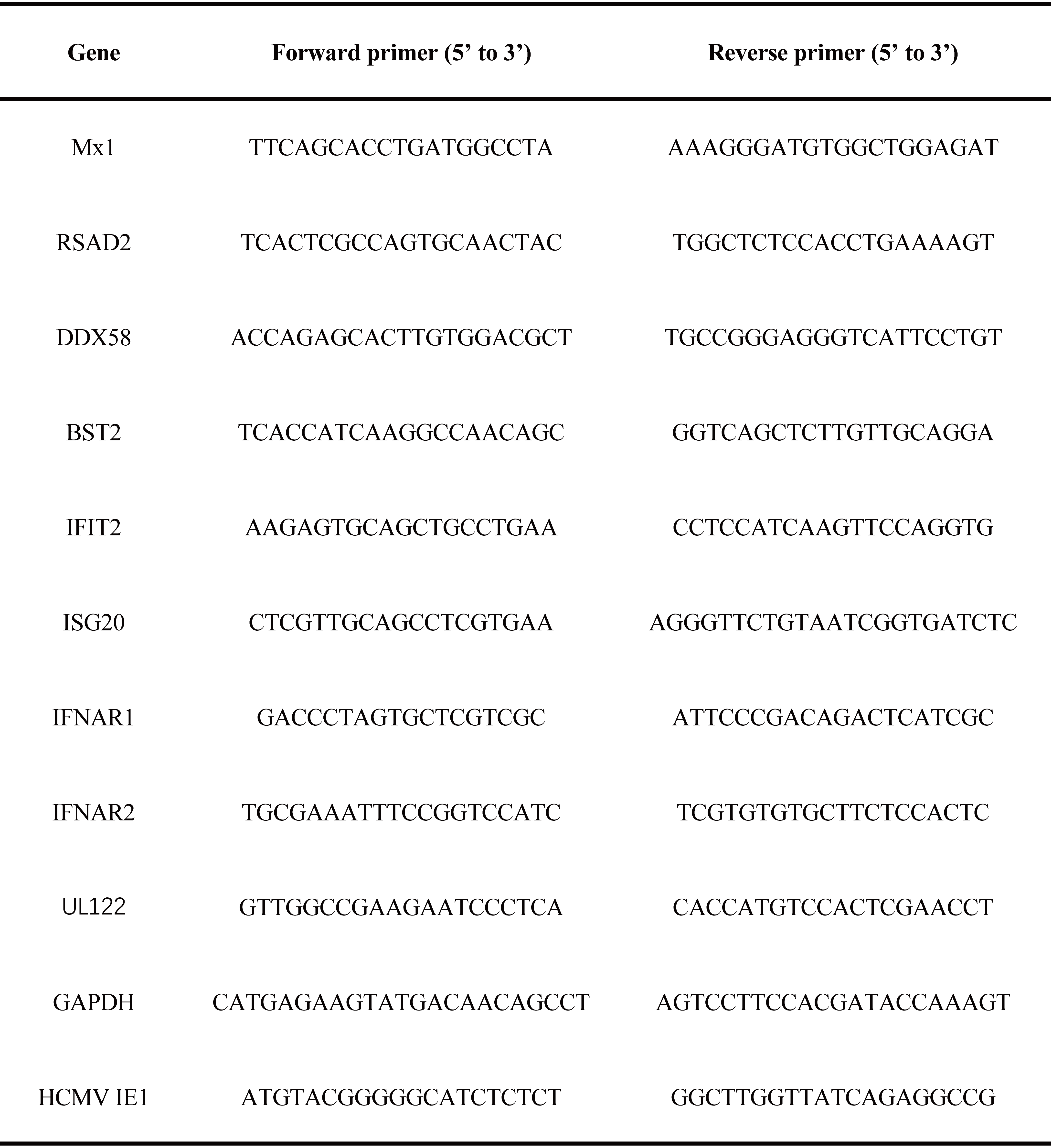


S-5 Primers used to examine miRNA


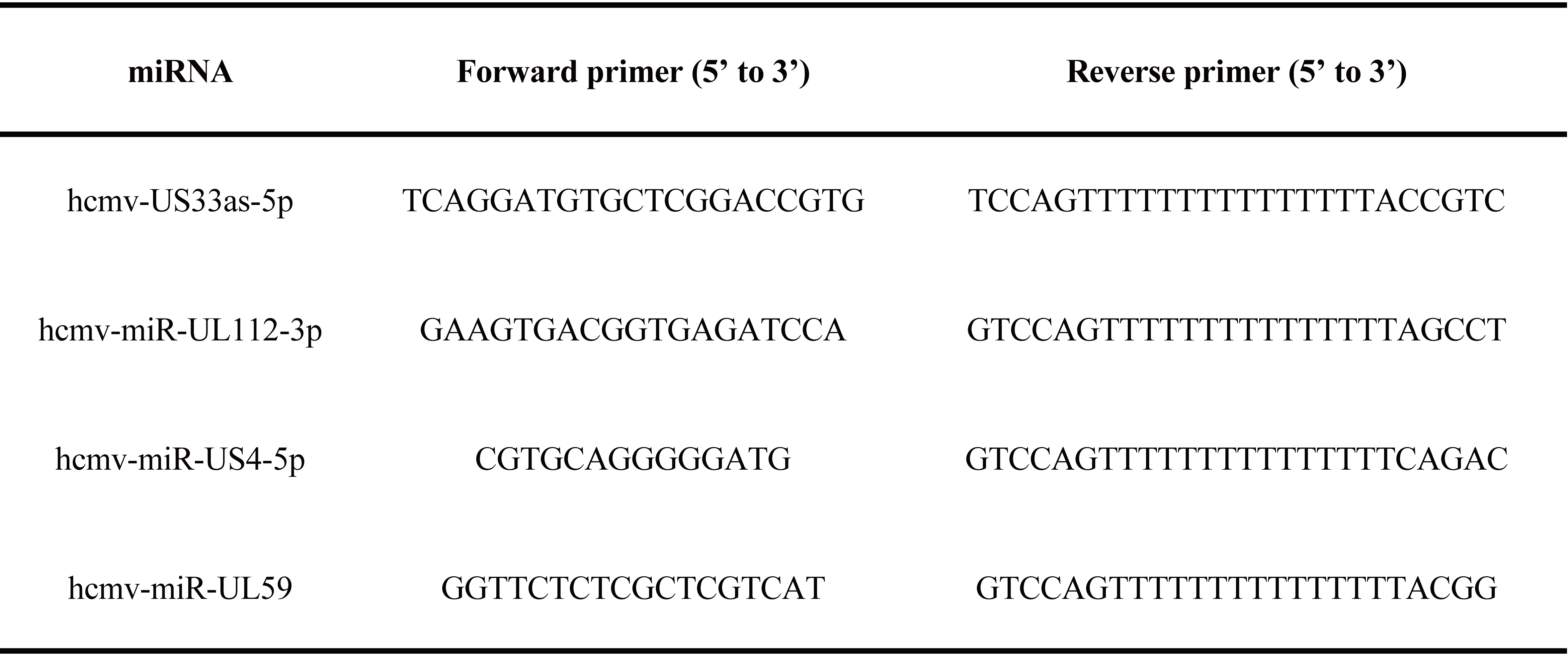

Supplement: Supplementary file 1 [file Data_Sheet_1.docx]
